# Supplementary material for: Non-line-of-sight snapshots and background mapping with an active corner camera
Source: Nat Commun. 2023 Jun 21;14:3677. doi: 10.1038/s41467-023-39327-2 (PMC10284852; doi:10.1038/s41467-023-39327-2)
Supplement: Supplementary file 1 — Supplementary Information [file 41467_2023_39327_MOESM1_ESM.pdf]

Supplementary Information for
Non-Line-of-Sight Snapshots and Background Mapping with an Active Corner Camera

Sheila Seidel, Hoover Rueda-Chacón, Iris Cusini, Federica Villa,
Franco Zappa, Christopher Yu, and Vivek K Goyal

Contents

Supplementary Note 1: Fast Forward Model Approximation **2**
Transient light transport modeling . . . 2
Fast computation of the response from a vertical planar facet . . . 2
Facet time bounds . . . 4

Supplementary Note 2: Inversion Algorithm **8**
Model and likelihood . . . 8
Estimation . . . 8
Combining estimates from subsequent frames . . . 12
Computing the vertices of occluded regions . . . 12

Supplementary Note 3: Experimental Considerations **13**
Acquisition Time Analysis . . . 13
Laser location . . . 13
SPAD location . . . 14

Supplementary Note 4: Additional Experimental Results **15**
Single-object demonstrations . . . 15
Two-object demonstration . . . 15
Robustness demonstrations . . . 17

Supplementary References **23**

Supplementary Note 1: Fast Forward Model Approximation

Transient light transport modeling

In our system, a pulsed laser and SPAD array are both pointed at the floor adjacent to an occluding edge. Take $\mathbf{p}_\mathrm{l}$ to be the position of the laser spot, $\mathbf{p}_\mathrm{f}$ to be a point in the area of the nth camera pixel $\mathcal{P}_n$, and $\mathbf{p}_\mathrm{s}$ to be a point on the hidden surface $\mathcal{S}$. The camera measurement rate integrated over the kth time bin at the nth spatial pixel is

$$\mathbf{s}^{n,k} = \int_{(k-1)\Delta_t}^{k\Delta_t} \int_{\mathcal{P}_n} \int_{\mathcal{S}} v(\mathbf{p}_\mathrm{s}, \mathbf{p}_\mathrm{f}) a(\mathbf{p}_\mathrm{s}) \frac{G(\mathbf{p}_\mathrm{s},\, \mathbf{p}_\mathrm{l},\, \mathbf{p}_\mathrm{f})}{\|\mathbf{p}_\mathrm{l}-\mathbf{p}_\mathrm{s}\|^2 \|\mathbf{p}_\mathrm{f}-\mathbf{p}_\mathrm{s}\|^2}\, w\!\left(t - t_0 - \frac{\|\mathbf{p}_\mathrm{l}-\mathbf{p}_\mathrm{s}\| + \|\mathbf{p}_\mathrm{f}-\mathbf{p}_\mathrm{s}\|}{c}\right) d\mathbf{p}_\mathrm{s}\, d\mathbf{p}_\mathrm{f}\, dt, \tag{1}$$

where $v(\mathbf{p}_\mathrm{s}, \mathbf{p}_\mathrm{f})$ is the visibility function defined in the main document, $a(\mathbf{p}_\mathrm{s})$ is the surface albedo at point $\mathbf{p}_\mathrm{s}$, $w(\cdot)$ is the pulsed waveform, Δ_t is the duration of a time bin, t_0 is the time the pulse hits the laser spot, and c is the speed of light. The Lambertian bidirectional reflectance distribution function (BRDF) factor $G(\cdot,\cdot,\cdot)$ is given by

$$G(\mathbf{p}_\mathrm{s},\, \mathbf{p}_\mathrm{l},\, \mathbf{p}_\mathrm{f}) = \cos\left(\measuredangle(\mathbf{p}_\mathrm{s}-\mathbf{p}_\mathrm{l}, \mathbf{n}_\mathrm{l})\right) \cos\left(\measuredangle(\mathbf{p}_\mathrm{l}-\mathbf{p}_\mathrm{s}, \mathbf{n}_\mathrm{s})\right) \cos\left(\measuredangle(\mathbf{p}_\mathrm{f}-\mathbf{p}_\mathrm{s}, \mathbf{n}_\mathrm{s})\right) \cos\left(\measuredangle(\mathbf{p}_\mathrm{s}-\mathbf{p}_\mathrm{f}, \mathbf{n}_\mathrm{f})\right), \tag{2}$$

where $\mathbf{n}_\mathrm{l}$, $\mathbf{n}_\mathrm{s}$, and $\mathbf{n}_\mathrm{f}$ are the surface normal vectors at points $\mathbf{p}_\mathrm{l}$, $\mathbf{p}_\mathrm{s}$, and $\mathbf{p}_\mathrm{f}$, respectively. When the camera pixel size is sufficiently small, (1) may be approximated by

$$\mathbf{s}^{n,k} \approx \Delta_\mathcal{P} \int_{(k-1)\Delta_t}^{k\Delta_t} \int_{\mathcal{S}} v(\mathbf{p}_\mathrm{s}, \bar{\mathbf{p}}_{\mathrm{f},n}) a(\mathbf{p}_\mathrm{s}) \frac{G(\mathbf{p}_\mathrm{s},\, \mathbf{p}_\mathrm{l},\, \bar{\mathbf{p}}_{\mathrm{f},n})}{\|\mathbf{p}_\mathrm{l}-\mathbf{p}_\mathrm{s}\|^2 \|\bar{\mathbf{p}}_{\mathrm{f},n}-\mathbf{p}_\mathrm{s}\|^2}\, w\!\left(t - t_0 - \frac{\|\mathbf{p}_\mathrm{l}-\mathbf{p}_\mathrm{s}\| + \|\bar{\mathbf{p}}_{\mathrm{f},n}-\mathbf{p}_\mathrm{s}\|}{c}\right) d\mathbf{p}_\mathrm{s}\, dt, \tag{3}$$

where $\Delta_\mathcal{P}$ is the area of the camera pixel and $\bar{\mathbf{p}}_{\mathrm{f},n}$ is the center of pixel n. When the pulse duration is short relative to the time bin length, we may replace $w(\cdot)$ by a Dirac impulse function $\delta(\cdot)$ scaled by a pulse intensity I to write

$$\mathbf{s}^{n,k} \approx \Delta_\mathcal{P} I \int_{(k-1)\Delta_t}^{k\Delta_t} \int_{\mathcal{S}} v(\mathbf{p}_\mathrm{s}, \bar{\mathbf{p}}_{\mathrm{f},n}) a(\mathbf{p}_\mathrm{s}) \frac{G(\mathbf{p}_\mathrm{s},\, \mathbf{p}_\mathrm{l},\, \bar{\mathbf{p}}_{\mathrm{f},n})}{\|\mathbf{p}_\mathrm{l}-\mathbf{p}_\mathrm{s}\|^2 \|\bar{\mathbf{p}}_\mathrm{f}-\mathbf{p}_\mathrm{s}\|^2}\, \delta\!\left(t - t_0 - \frac{\|\mathbf{p}_\mathrm{l}-\mathbf{p}_\mathrm{s}\| + \|\bar{\mathbf{p}}_{\mathrm{f},n}-\mathbf{p}_\mathrm{s}\|}{c}\right) d\mathbf{p}_\mathrm{s}\, dt. \tag{4}$$

Fast computation of the response from a vertical planar facet

Our inversion algorithm is based on a planar facet model of the hidden scene. Thus, we are interested in quickly computing the response of a vertical planar facet resting on the floor, shown in grey in Supplementary Figure 1a. The method we develop here achieves a speed gain factor of about 150 over a direct numerical integration with similar accuracy.

We define a coordinate system (different than in the main document) so that the laser spot $\mathbf{p}_\mathrm{l}$ and pixel center $\bar{\mathbf{p}}_{\mathrm{f},n}$ are placed a distance m apart along the y-axis equidistant from the origin, as marked in Supplementary Figure 1a. For any $d > m$, points $\mathbf{p}_\mathrm{l}$ and $\bar{\mathbf{p}}_{\mathrm{f},n}$ form the foci of an ellipsoid that contains all points $\mathbf{p}_\mathrm{s}$ in the scene with round-trip travel distance $d = \|\mathbf{p}_\mathrm{l}-\mathbf{p}_\mathrm{s}\| + \|\bar{\mathbf{p}}_{\mathrm{f},n}-\mathbf{p}_\mathrm{s}\|$. The intersection of that ellipsoid with the planar facet is an ellipse segment, as shown in red in Supplementary Figure 1a. Now consider the integration in (4). As t increases, the d value matching the shift of the Dirac grows and the corresponding ellipse intersection expands. Over the duration of the time bin, the integral represents the light returning from the dark grey annulus, between the red and blue segments, shown in Supplementary Figure 1a. In [1], $\mathbf{p}_\mathrm{l}$ and $\mathbf{p}_\mathrm{f}$ are treated as being at the origin, so that the ellipsoid reduces to a sphere and the planar intersection reduces from an ellipse to a circle; the double integral in (4) is converted to polar coordinates, resulting in an approximate, closed-form solution to (4). In this work, since the spatial diversity of our sensor array is essential, we cannot use this approximation. Instead, we develop a quick computation of the rates due to a vertical facet under the more general elliptical model.

Consider the coordinate system in Supplementary Figure 1a with laser position $\mathbf{p}_\mathrm{l}$ and pixel center $\bar{\mathbf{p}}_{\mathrm{f},n}$. The ellipsoid corresponding to round-trip travel distance d may be written as

$$1 = \frac{x^2}{a_\mathrm{e}^2} + \frac{y^2}{b_\mathrm{e}^2} + \frac{z^2}{c_\mathrm{e}^2}, \tag{5}$$

where $a_\mathrm{e} = c_\mathrm{e} = \sqrt{(d/2)^2 - (m/2)^2}$ and $b_\mathrm{e} = d/2$. The planar facet is contained in a plane that may be described by normal vector $\mathbf{n}_\mathrm{s}$ and point $\mathbf{q}$. The intersection of this plane and the ellipsoid in (5) is an ellipse that may be written in translational form [2] in a new $[r,\, s,\, u]^\top$ coordinate system:

$$1 = \frac{r^2}{A^2} + \frac{s^2}{B^2}. \tag{6}$$

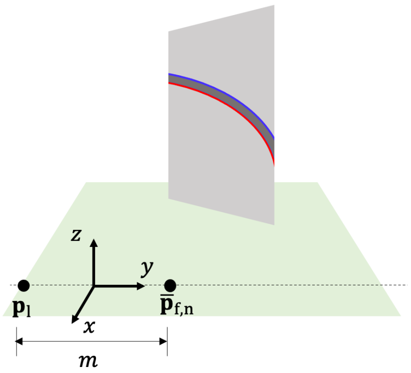

(a) Laser spot $\mathbf{p}_\mathrm{l}$ and pixel center $\bar{\mathbf{p}}_{\mathrm{f},n}$ form the focii of an ellipsoid that contains points with equal round-trip travel time. The intersection of an ellipsoid and a planar facet (light gray) is an ellipse segment (ex. red and blue lines). The occluding edge is not pictured.

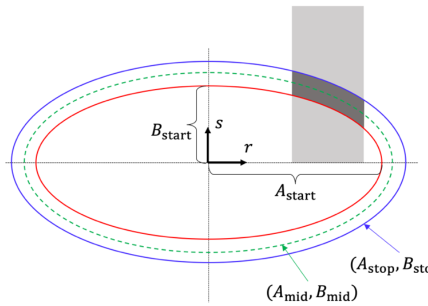

(b) In the rsu coordinate system, the ellipse intersection may be written in translational form with major and minor axis lengths A and B. Axis lengths $(A_\mathrm{start}, B_\mathrm{start})$, $(A_\mathrm{mid}, B_\mathrm{mid})$, and $(A_\mathrm{stop}, B_\mathrm{stop})$ correspond to ellipses formed by the start, middle, and stop times of a time bin. The dark gray annulus is the region of the facet illuminated over the course of a time bin.

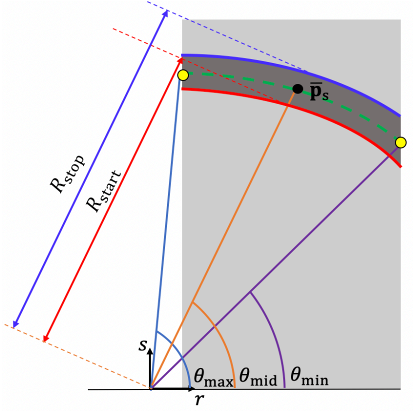

(c) The facet response at a given time bin is the sum of the integrand over the dark gray annulus. The angle θ_mid bisects the minimum and maximum angles θ_mid and θ_max. Ranges R_start and R_stop are the ranges of ellipses $(A_\mathrm{start}, B_\mathrm{start})$ and $(A_\mathrm{stop}, B_\mathrm{stop})$ at angle θ_mid.

Supplementary Figure 1: The ellipsoid-plane intersection shown in (a) is shown as an ellipse written in translational form in (b). The response of a facet in a given time bin can be thought of as a polar integral over the dark grey region in (c).

If $\mathbf{q}$ is chosen to be interior to the ellipsoid, the procedure in [2] may be used to find the new $[r, s, u]$ coordinate system and provides formulae for A and B given $\mathbf{n}_\mathrm{s}$, $\mathbf{q}$, and the ellipsoid parameters $(a_\mathrm{e}, b_\mathrm{e}, c_\mathrm{e})$. Supplementary Figure 1b shows ellipses corresponding to the start (parameters: A_start and B_start), middle (parameters: A_mid and B_mid), and stop (parameters: A_stop and B_stop) times of a time bin in the rs-plane with the facet in grey. The double integral in (4) sums the integrand over the dark grey annulus. This double integral can be formulated as integration in polar coordinates over a region in the rs-plane as shown in Supplementary Figure 1c. For computational speed, we approximate this integral as

$$\mathbf{s}^{n,k} \approx \Delta_\mathcal{P} I \Delta_\mathrm{arc}^{n,k} a \frac{G(\bar{\mathbf{p}}_\mathrm{s}, \mathbf{p}_\mathrm{l}, \bar{\mathbf{p}}_{\mathrm{f},n})}{\|\mathbf{p}_\mathrm{l} - \bar{\mathbf{p}}_\mathrm{s}\|^2 \|\bar{\mathbf{p}}_{\mathrm{f},n} - \bar{\mathbf{p}}_\mathrm{s}\|^2}, \tag{7}$$

where $\Delta_\mathrm{arc}^{n,k}$ is the annulus area. The point $\bar{\mathbf{p}}_\mathrm{s}$ is on the ellipse corresponding to the middle of the time bin (i.e., with parameters A_mid and B_mid) at polar angle $\theta_\mathrm{mid} = (\theta_\mathrm{min} + \theta_\mathrm{max})/2$. Note that we have replaced $a(\mathbf{p}_\mathrm{s})$ with a under the assumption that the facet has uniform albedo. The area $\Delta_\mathrm{arc}^{n,k}$ is approximated as a fraction of a circular annulus:

$$\Delta_\mathrm{arc}^{n,k} \approx \frac{\theta_\mathrm{max} - \theta_\mathrm{min}}{2\pi}(\pi R_\mathrm{stop}^2 - \pi R_\mathrm{start}^2) = \frac{1}{2}(\theta_\mathrm{max} - \theta_\mathrm{min})(R_\mathrm{stop}^2 - R_\mathrm{start}^2). \tag{8}$$

Ellipse radii at polar angle $\theta = \theta_\mathrm{mid}$: R_start and R_stop, marked in Supplementary Figure 1c, may be computed using

$$R(\theta, A, B) = \frac{AB}{\sqrt{B^2 \cos^2\theta + A^2 \sin^2\theta}}. \tag{9}$$

In practice, when a facet is wide, the extent of the annulus may not be well described by a single central point $\bar{\mathbf{p}}_\mathrm{s}$. When the distance d_annulus between the edges of the annulus, marked with yellow dots in Supplementary Figure 1c, is greater than parameter d_max, the annulus is divided into smaller annulus segments that each satisfy $d_\mathrm{annulus} < d_\mathrm{max}$. The full procedure for computing the facet response at each of the N camera pixels and K time bins is outlined in Algorithm 1.

Supplementary Figure 2 compares facet responses computed using Algorithm 1 (solid line) with those computed using a slower high-fidelity simulation tool based on numerical integration (dashed line with markers) for a person-sized facet at four different azimuthal rotation angles. Different colored curves denote rates computed at different array pixels. Note that in all four cases, our fast forward model computation closely matches the higher-fidelity simulator. In Supplementary Figure 3, we show results for a shorter facet, similar in height to the child-sized mannequin we use in our experiments. The shorter facet also exhibits close match between Algorithm 1 and the higher-fidelity simulation. Supplementary Figure 4 shows $\|\mathbf{s}_\mathrm{exact} - \mathbf{s}_\mathrm{fast}\|_1 / \|\mathbf{s}_\mathrm{exact}\|_1$ at different

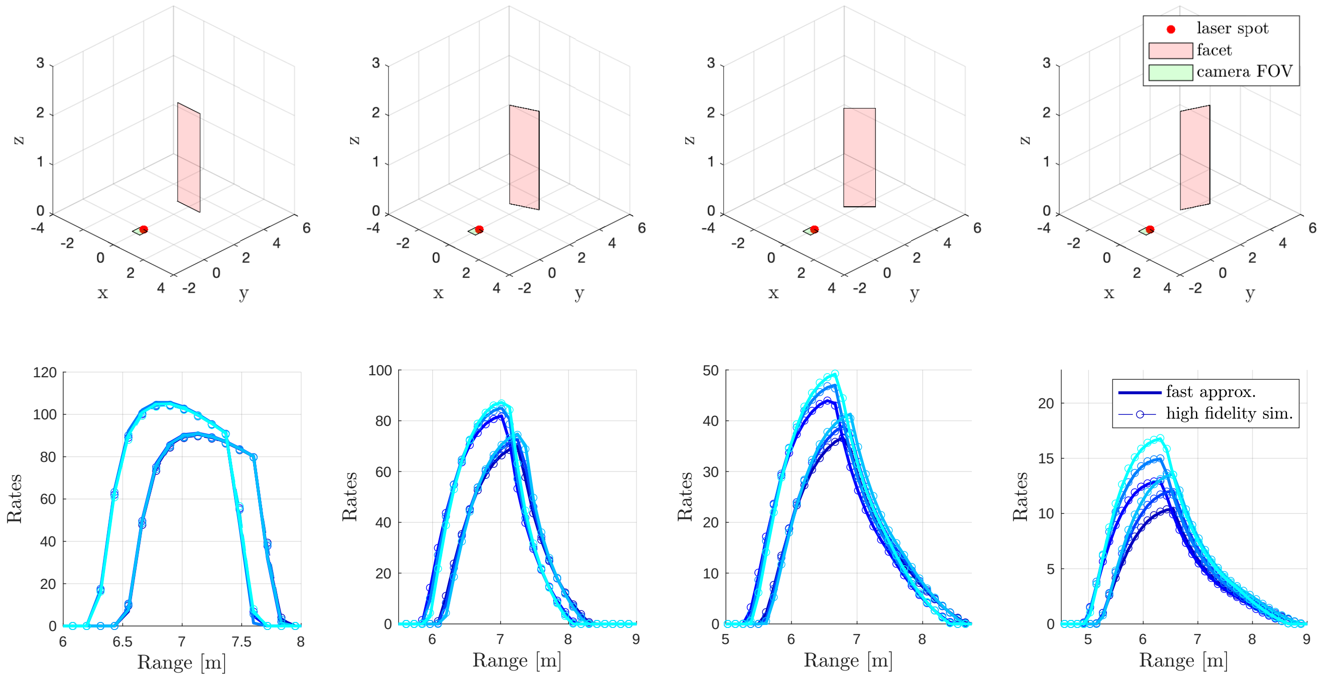

(a) rotation $= 0$, $\frac{\|\mathbf{s}_{\text{exact}} - \mathbf{s}_{\text{fast}}\|_1}{\|\mathbf{s}_{\text{exact}}\|_1} = 0.0248$

(b) rotation $= \pi/8$, $\frac{\|\mathbf{s}_{\text{exact}} - \mathbf{s}_{\text{fast}}\|_1}{\|\mathbf{s}_{\text{exact}}\|_1} = 0.0074$

(c) rotation $= 2\pi/8$, $\frac{\|\mathbf{s}_{\text{exact}} - \mathbf{s}_{\text{fast}}\|_1}{\|\mathbf{s}_{\text{exact}}\|_1} = 0.0092$

(d) rotation $= 3\pi/8$, $\frac{\|\mathbf{s}_{\text{exact}} - \mathbf{s}_{\text{fast}}\|_1}{\|\mathbf{s}_{\text{exact}}\|_1} = 0.0118$

Supplementary Figure 2: A comparison of Algorithm 1 to conventional numerical integration for a person-sized facet at various tilt angles with a width of 0.75 m and height of 2 m. The first row of plots shows the position of the SPAD FOV, laser spot, and the facet. The bottom row shows computed rates at select pixels for each method. The SPAD FOV is 0.5 $\times$ 0.5 m. (a) 0 radians; (b) $\pi/8$ radians; (c) $2\pi/8$ radians; (d) $3\pi/8$ radians.

points in space for a vertical facet facing the origin. Here, $\mathbf{s}_{\text{fast}}$ is the vector of rates computed using Algorithm 1 and $\mathbf{s}_{\text{exact}}$ is generated using the higher-fidelity simulation. Although the shorter facet (a) has more error (i.e., larger $\|\mathbf{s}_{\text{exact}} - \mathbf{s}_{\text{fast}}\|_1 / \|\mathbf{s}_{\text{exact}}\|_1$) than the taller facet (b), both have relatively small error. In our experimental demonstrations, we use a small scaled-down room with objects similar in size to the one tested in Supplementary Figure 4a. Although our experimental results in Supplementary Note 4 are achieved using a scaled down setup, Supplementary Figure 4b suggests that the forward model computation in Algorithm 1 is even more accurate in a larger, more life-like setting.

Facet time bounds

Take $t_{\min}$ and $t_{\max}$ to be the smallest and largest arrival times due to the facet, corresponding to round-trip travel distances $d_{\min}$ and $d_{\max}$. We seek $d_{\min}$ and $d_{\max}$ in order to compute rates due to the facet at all affected time bins. To simplify the geometry, we assume we are only interested in vertical rectangular facets resting on the floor. Under this assumption, the part of the facet with the shortest round-trip travel distance is somewhere along its bottom edge. The two bottom vertices of the planar facet $\mathbf{v}_1$ and $\mathbf{v}_2$ are contained in the xy-plane (i.e., the ground plane), as shown in Supplementary Figure 5. When $\mathbf{v}_2(1) \neq \mathbf{v}_1(1)$, the line that runs along the bottom of the facet, connecting the two lowest vertices ($\mathbf{v}_1$ and $\mathbf{v}_2$), is given by

$$y = m_{\text{line}} x + b_{\text{line}}, \tag{10}$$

where

$$m_{\text{line}} = \frac{\mathbf{v}_2(2) - \mathbf{v}_1(2)}{\mathbf{v}_2(1) - \mathbf{v}_1(1)}, \tag{11a}$$

$$b_{\text{line}} = -\mathbf{v}_1(1) m_{\text{line}} + \mathbf{v}_1(2). \tag{11b}$$

Algorithm 1 Fast computation of rates at the SPAD array due to a vertical planar facet resting on the floor

for each $n = [1, 2, \ldots, N]$ **do** ▷ loop through camera pixels
  find indices of first and last affected time bins k_{min} and k_{max} according to (21)
  for each $k = [k_{\text{min}}, k_{\text{min}} + 1, \ldots, k_{\text{max}}]$ **do** ▷ loop through affected time bins
    find rsu coordinate system and compute ellipse parameters $(A_{\text{mid}}, B_{\text{mid}})$ and $(A_{\text{stop}}, B_{\text{stop}})$ using [2]
    if $k == k_{\text{min}}$ **do**
      compute ellipse parameters $(A_{\text{start}}, B_{\text{start}})$
    end if
    find θ_{min} and θ_{max} by finding intersections of ellipse $(A_{\text{mid}}, B_{\text{mid}})$ with facet edges
    compute $\theta_{\text{mid}} = \frac{1}{2}(\theta_{\text{min}} + \theta_{\text{max}})$
    compute R_{start} and R_{stop} using (9) with arguments $(A_{\text{start}}, B_{\text{start}})$, $(A_{\text{stop}}, B_{\text{stop}})$, and θ_{mid}
    compute approximate facet response in kth time bin at ith pixel using (7) and (8)
    $(A_{\text{start}}, B_{\text{start}}) \leftarrow (A_{\text{stop}}, B_{\text{stop}})$ ▷ next time bin starts with end of this one
  end for each
end for each

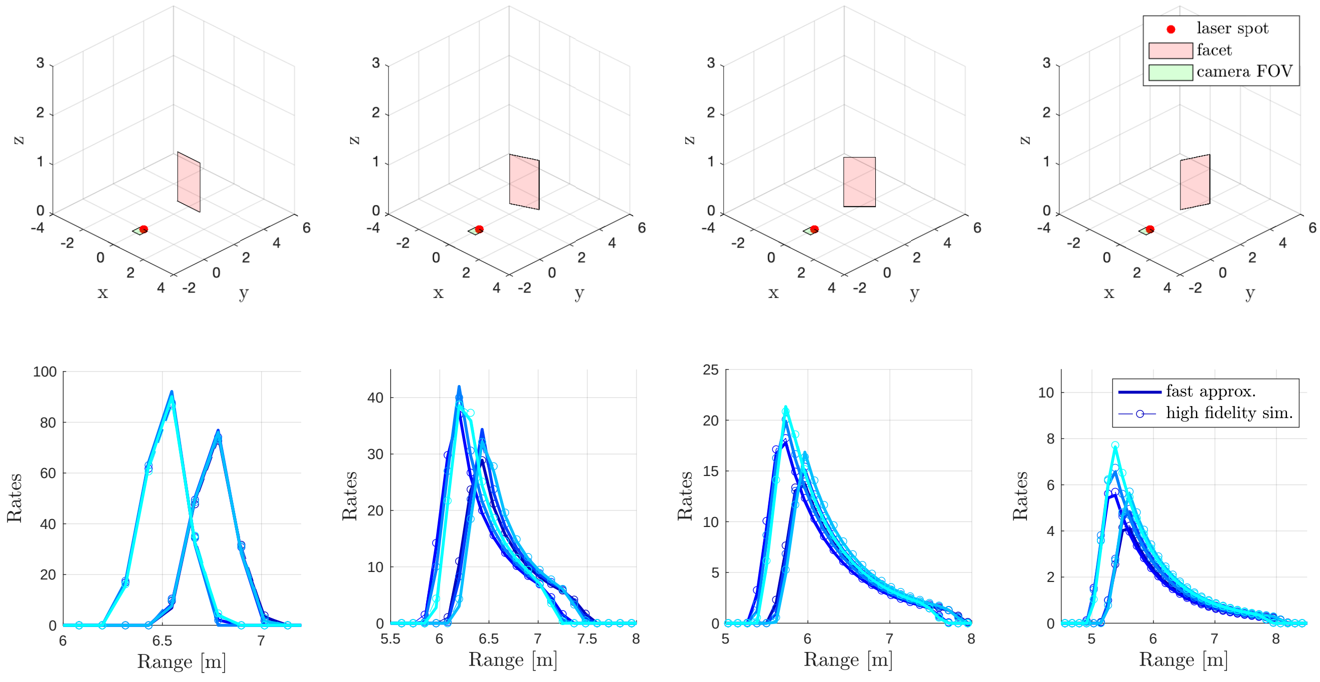

(a) rotation $= 0$, $\frac{\|\mathbf{s}_{\text{exact}} - \mathbf{s}_{\text{fast}}\|_1}{\|\mathbf{s}_{\text{exact}}\|_1} = 0.0775$

(b) rotation $= \pi/8$, $\frac{\|\mathbf{s}_{\text{exact}} - \mathbf{s}_{\text{fast}}\|_1}{\|\mathbf{s}_{\text{exact}}\|_1} = 0.0300$

(c) rotation $2\pi/8$, $\frac{\|\mathbf{s}_{\text{exact}} - \mathbf{s}_{\text{fast}}\|_1}{\|\mathbf{s}_{\text{exact}}\|_1} = 0.0316$

(d) rotation $= 3\pi/8$, $\frac{\|\mathbf{s}_{\text{exact}} - \mathbf{s}_{\text{fast}}\|_1}{\|\mathbf{s}_{\text{exact}}\|_1} = 0.0338$

Supplementary Figure 3: A comparison of Algorithm 1 to conventional numerical integration for a child-sized facet at various tilt angles with a width of 0.75 m and a height of 1 m. The first row of plots shows the position of the SPAD FOV, laser spot, and the facet. The bottom row shows computed rates at select pixels for each method. The SPAD FOV is 0.5×0.5 m. (a) 0 radians; (b) $\pi/8$ radians; (c) $2\pi/8$ radians; (d) $3\pi/8$ radians.

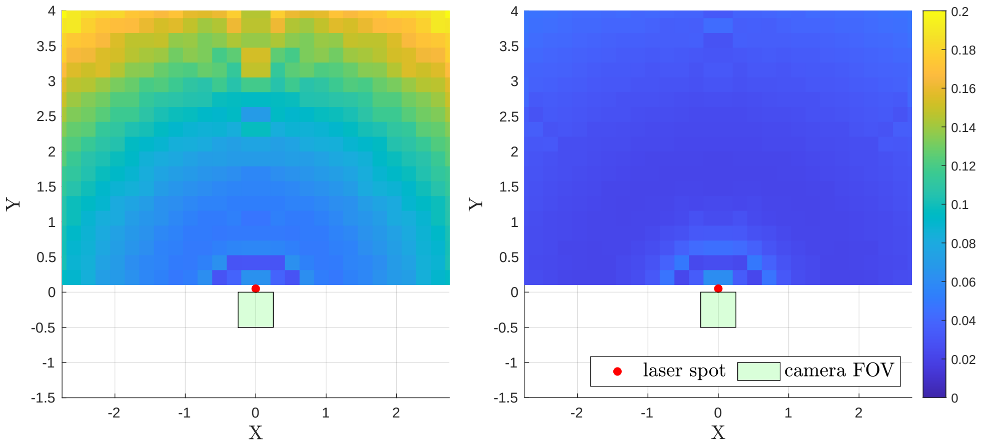

(a) Facet is 1m tall and 1m wide

(b) Facet is 2m tall and 1m wide

Supplementary Figure 4: Relative error metric $\|\mathbf{s}_{\text{exact}} - \mathbf{s}_{\text{fast}}\|_1 / \|\mathbf{s}_{\text{exact}}\|_1$ plotted for different facet positions in space for a 1 m tall facet (a) and a 2 m tall facet (b). All facets face the occluding edge.

The intersection of the ground plane with the ellipsoid in (5) is an ellipse ("Ellipse Ground") (not to be confused with the ellipse in the rs-plane of (6)), with foci at $[0,\ m/2]$ and $[0,\ -m/2]$. The equation for this ellipse in the xy-plane is

$$\frac{x^2}{A_{\text{ground}}^2} + \frac{y^2}{B_{\text{ground}}^2} = 1, \tag{12}$$

where

$$A_{\text{ground}}(d_{\text{int}}) = \sqrt{\left(\frac{d_{\text{int}}}{2}\right)^2 - \left(\frac{m}{2}\right)^2}, \tag{13a}$$

$$B_{\text{ground}}(d_{\text{int}}) = \frac{d_{\text{int}}}{2}. \tag{13b}$$

When the condition

$$b_{\text{line}}^2 = A_{\text{ground}}^2 m_{\text{line}}^2 + B_{\text{ground}}^2 \tag{14}$$

is met, Ellipse Ground (12) and the line in (10) intersect at a single point, rather than two, corresponding to round-trip travel distance d_{int}. Substituting (13a) and (13b) into (14) and solving for d_{int} yields

$$d_{\text{int}} = 2\sqrt{\frac{b_{\text{line}}^2 + \left(\frac{m_{\text{line}} m}{2}\right)^2}{m_{\text{line}}^2 + 1}}. \tag{15}$$

We use d_{int} in (13a) and (13b) to solve for A_{ground} and B_{ground} and find the corresponding single point of intersection by solving (10) and (12) for $(x_{\text{int}}, y_{\text{int}})$:

$$x_{\text{int}} = \frac{-A_{\text{ground}}^2 m_{\text{line}} b_{\text{line}}}{B_{\text{ground}}^2 + A_{\text{ground}}^2 m_{\text{line}}^2}, \tag{16a}$$

$$y_{\text{int}} = m_{\text{line}} x_{\text{int}} + b_{\text{line}}. \tag{16b}$$

If the point $(x_{\text{int}}, y_{\text{int}})$ is on the line segment between $\mathbf{v}_1$ and $\mathbf{v}_2$, then the shortest round-trip travel time from $\mathbf{p}_{\text{l}}$ to the planar facet and back to $\mathbf{p}_{\text{f}}$ is $d_{\text{min}} = d_{\text{int}}$. If the point $(x_{\text{int}}, y_{\text{int}})$ is *not* on the line segment between $\mathbf{v}_1$ and $\mathbf{v}_2$, then the point on the line segment that is closest to the point $(x_{\text{int}}, y_{\text{int}})$ is the point with the shortest round-trip travel time from $\mathbf{p}_{\text{l}}$ to the facet and back to $\mathbf{p}_{\text{f}}$. This is one of the bottom facet vertices $\mathbf{v}_1$ or $\mathbf{v}_2$:

$$d_{\text{min}} = \min\left\{ \|\mathbf{p}_{\text{l}} - \mathbf{v}_1\| + \|\mathbf{p}_{\text{f}} - \mathbf{v}_1\|,\ \|\mathbf{p}_{\text{l}} - \mathbf{v}_2\| + \|\mathbf{p}_{\text{f}} - \mathbf{v}_2\| \right\}. \tag{17}$$

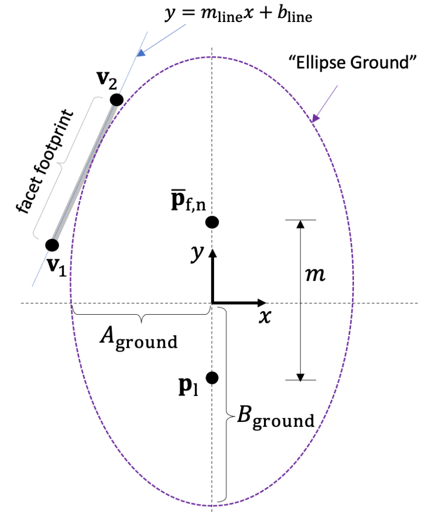

Supplementary Figure 5: With both $\mathbf{p}_{\text{l}}$ and $\bar{\mathbf{p}}_{\text{f},n}$ in the ground plane, and the vertical hidden facet resting on the ground, the part of the facet with the shortest round-trip travel time is also on the ground. Regions with equal round-trip travel time from $\mathbf{p}_{\text{l}}$, to the hidden scene, and back to $\bar{\mathbf{p}}_{\text{f},n}$ form ellipsoids. The intersection of this ellipsoid and the ground plane is an ellipse (purple). The shortest round-trip travel time that causes this ellipse to encounter the bottom of the facet (i.e., the line segment between $\mathbf{v}_1$ and $\mathbf{v}_2$) is the earliest time of arrival due to the facet.

The longest round-trip travel time $d_{\max}$ corresponds to the furthest of the top two vertices ($\mathbf{v}_3$ and $\mathbf{v}_4$):

$$d_{\max} = \max\left\{\|\mathbf{p}_{\text{l}} - \mathbf{v}_3\| + \|\mathbf{p}_{\text{f}} - \mathbf{v}_3\|,\ \|\mathbf{p}_{\text{l}} - \mathbf{v}_4\| + \|\mathbf{p}_{\text{f}} - \mathbf{v}_4\|\right\}. \tag{18}$$

In the special case where $\mathbf{v}_2(1) = \mathbf{v}_1(1)$, we recall that $\mathbf{p}_{\text{l}}$ and $\mathbf{p}_{\text{f}}$ are equally spaced from the origin along the y-axis. In this case, if $\operatorname{sign}(\mathbf{v}_1(2)) \neq \operatorname{sign}(\mathbf{v}_2(2))$, we know that the point on the line segment from $\mathbf{v}_1$ to $\mathbf{v}_2$ with the shortest round-trip travel time is at $[\mathbf{v}_2(1),\, 0,\, \mathbf{v}_2(3)]^\top$ and

$$d_{\min} = \left\|\mathbf{p}_{\text{l}} - [\mathbf{v}_2(1),\, 0,\, \mathbf{v}_2(3)]^\top\right\| + \left\|\mathbf{p}_{\text{f}} - [\mathbf{v}_2(1),\, 0,\, \mathbf{v}_2(3)]^\top\right\|. \tag{19}$$

When $\operatorname{sign}(\mathbf{v}_1(2)) = \operatorname{sign}(\mathbf{v}_2(2))$, the shortest round-trip travel time is to the vertex $\mathbf{v}_1$ or $\mathbf{v}_2$ with the smallest magnitude y-coordinate:

$$d_{\min} = \min\left\{\|\mathbf{p}_{\text{l}} - \mathbf{v}_1\| + \|\mathbf{p}_{\text{f}} - \mathbf{v}_1\|,\ \|\mathbf{p}_{\text{l}} - \mathbf{v}_2\| + \|\mathbf{p}_{\text{f}} - \mathbf{v}_2\|\right\}. \tag{20}$$

The indices of the first and last affected time bins are

$$k_{\min} = \operatorname{floor}(d_{\min}/(c\Delta_t)), \tag{21a}$$
$$k_{\max} = \operatorname{floor}(d_{\max}/(c\Delta_t)). \tag{21b}$$

Supplementary Note 2: Inversion Algorithm

In this work, rather than estimate a complicated distributed scene from a single measurement frame, we seek a robust reconstruction of *change* from a reference measurement in the hidden scene. The reference measurement includes light returning from hidden- and visible-side stationary scene content. When an object enters the foreground of the hidden scene, the new measurement changes to include added rates due to the foreground object as well as a rate reduction due to the object's occlusion of the background. At each new frame, we use these changes to reconstruct the moving object as well as the stationary background behind it. As an object traverses the hidden scene, these background segments accumulate to form a reconstruction of the stationary hidden scene.

Model and likelihood

When objects move into the hidden scene, the camera measurement at the nth spatial pixel and the kth time bin is Poisson distributed,

$$\mathbf{x}^{n,k} \sim \text{Poisson}(\mathbf{b}^{n,k} + \mathbf{s}_{\text{fg}}^{n,k}(\boldsymbol{\psi}_{\text{fg}}) - \mathbf{s}_{\text{oc}}^{n,k}(\boldsymbol{\psi}_{\text{fg}}, \boldsymbol{\psi}_{\text{oc}})), \tag{22}$$

where $\mathbf{b} \in \mathbb{R}^{N \times K}$ is the rates due to stationary scenery, $\mathbf{s}_{\text{fg}} \in \mathbb{R}^{N \times K}$ is the response of the objects, and $\mathbf{s}_{\text{oc}} \in \mathbb{R}^{N \times K}$ is the response of the occluded background region, before the objects enter. We assume that we have observed the floor for long enough that $\mathbf{b}$ is approximately known.[1] Vectors $\boldsymbol{\psi}_{\text{fg}}$ and $\boldsymbol{\psi}_{\text{oc}}$ contain parameters that describe the foreground objects and corresponding occluded background regions, all of which are modeled as vertical, planar, rectangular facets that face the occluding edge. The foreground facets have parameters

$$\boldsymbol{\psi}_{\text{fg}} = \{(\boldsymbol{\theta}^m, a_{\text{fg}}^m, r_{\text{fg}}^m, h^m), \quad m = 1, \ldots, M\}, \tag{23}$$

where facet m has albedo a_{fg}^m, range r_{fg}^m, and height h^m; the number of foreground facets is M. Angles $\boldsymbol{\theta}^m = (\theta_{\text{min}}^m, \theta_{\text{max}}^m)$ are the minimum and maximum polar angles of the foreground facet m, measured around the occluding edge in the plane of the floor. The occluded regions have parameters

$$\boldsymbol{\psi}_{\text{oc}} = \{(a_{\text{oc}}^m, r_{\text{oc}}^m), \quad m = 1, \ldots, M\}, \tag{24}$$

where occluded background region m has albedo a_{oc}^m and range r_{oc}^m. As described in ***Computing the vertices of occluded regions*** below, the height of the occluded region depends upon its range r_{oc} and on the range r_{fg} and height h of the foreground facet. Given parameters $\boldsymbol{\psi}_{\text{fg}}$ and $\boldsymbol{\psi}_{\text{oc}}$, the procedure outlined in Algorithm 1 may be used to quickly compute $\mathbf{s}_{\text{fg}}$ and $\mathbf{s}_{\text{oc}}$.

Because all the Poisson random variables in (22) are independent, the likelihood of the measurement vector $\mathbf{x}$ given parameters $\boldsymbol{\psi}_{\text{fg}}$ and $\boldsymbol{\psi}_{\text{oc}}$ is the product

$$f(\mathbf{x} \,|\, \boldsymbol{\psi}_{\text{fg}}, \boldsymbol{\psi}_{\text{oc}}) = \prod_{n=1}^{N} \prod_{k=1}^{K} \frac{\Big(\mathbf{b}^{n,k} + \mathbf{s}_{\text{fg}}^{n,k}(\boldsymbol{\psi}_{\text{fg}}) - \mathbf{s}_{\text{oc}}^{n,k}(\boldsymbol{\psi}_{\text{fg}}, \boldsymbol{\psi}_{\text{oc}})\Big)^{\mathbf{x}^{n,k}} \exp\Big(-\Big(\mathbf{b}^{n,k} + \mathbf{s}_{\text{fg}}^{n,k}(\boldsymbol{\psi}_{\text{fg}}) - \mathbf{s}_{\text{oc}}^{n,k}(\boldsymbol{\psi}_{\text{fg}}, \boldsymbol{\psi}_{\text{oc}})\Big)\Big)}{\mathbf{x}^{n,k}!}. \tag{25}$$

Estimation

Our inversion algorithm, outlined in Supplementary Figure 6 and Supplementary Figure 7, estimates the parameters $\boldsymbol{\psi}_{\text{fg}}$ and $\boldsymbol{\psi}_{\text{oc}}$ for each measurement frame where motion has occurred.

Parameter initialization. Supplementary Figure 6 covers the parameter initialization procedure. Rates due to stationary scenery on visible and hidden sides of the occluding wall (red) are estimated from an initial reference measurement. In the pre-processing stage, a difference frame (blue) $\mathbf{y}$ is computed by subtracting the estimated background rates (red) $\mathbf{b}$ from the new measurement frame (green) $\mathbf{x}_{\text{t}}$: $\mathbf{y} = \mathbf{x}_{\text{t}} - \mathbf{b}$. For each time index k, this difference frame is spatially integrated (across all spatial pixels in the SPAD array) to create the collapsed profile of photon counts versus range, $\mathbf{y}_{\text{SI}}$ (orange):

$$\mathbf{y}_{\text{SI}}^k = \sum_{n=1}^{N} \mathbf{y}^{n,k}. \tag{26}$$

The light travel distances corresponding to the maximum and minimum values of $\mathbf{y}_{\text{SI}}$ are halved and used to initialize parameters r_{fg} and r_{oc}. The time bin indices K_{fg} of the foreground object are selected according to

$$K_{\text{fg}} = \left\{k \;\middle|\; k < p,\;\; \mathbf{y}_{\text{SI}}^k \geq \beta_{\text{time}} \max(\mathbf{y}_{\text{SI}})\right\}, \tag{27}$$

[1] It has also been shown that taking the median at each spatial pixel and time bin over a sequence of measurement frames produces a useful proxy for $\mathbf{b}$, even when objects are moving in the hidden scene [3,4].

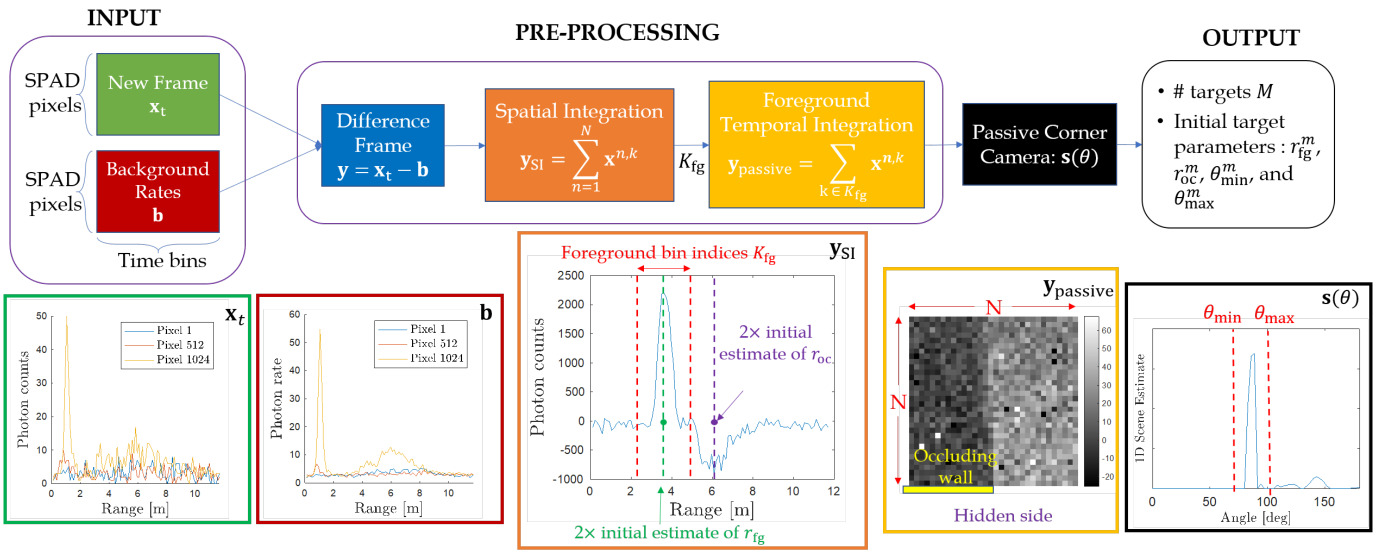

Supplementary Figure 6: Outline of the parameter initialization procedure.

where p is the index of the minimum entry of $\mathbf{y}_{\text{SI}}$ and $\beta_{\text{time}} < 1$ is a tuning parameter used to set the threshold. For each pixel n, temporal integration (yellow) of $\mathbf{y}_t$ is performed over the foreground time bin indices to form the equivalent of a passive measurement:

$$\mathbf{y}_{\text{passive}}^{n} = \sum_{k \in K_{\text{fg}}} \mathbf{y}^{n,k}. \tag{28}$$

An example of $\mathbf{y}_{\text{passive}}$ for a single-object scenario, with a clear penumbra pattern, is pictured in the yellow box in Supplementary Figure 6. This 'passive' measurement $\mathbf{y}_{\text{passive}}$ is fed into the one-dimensional passive corner camera algorithm of [5][2] to produce a one-dimensional profile of the hidden scene $\mathbf{s}_\theta \in \mathbb{R}^Q$ as a function of azimuthal angle θ around the corner, where Q is the number of angular bins in our discrete representation of the 1D hidden scene. In all results presented here, $Q = 64$. An example of $\mathbf{s}_\theta$ is shown in the black box in Supplementary Figure 6, where a sharp peak is visible at the object location around $\theta = \pi/2$. The azimuthal resolving power of the vertical edge makes this representation of our data well suited for counting the number of objects M moving in our hidden scene [6]. We compare $\mathbf{s}_\theta$ to a threshold $(\beta_\theta/Q)\sum_{q=1}^{Q} \mathbf{s}_\theta^q$, where β_θ is a tuning parameter. Each interval where $\mathbf{s}_\theta$ is above the threshold is considered to be a single object. The angles of the first and last threshold crossing for each object are used to initialize parameters $\theta_{\min}$ and $\theta_{\max}$.

Parameter estimation. Supplementary Figure 7 outlines the parameter estimation and post-processing steps. Parameter estimation is performed using the Metropolis–Hastings (MH) algorithm, a type of Markov chain Monte Carlo (MCMC) method, and the likelihood in (25). Note that although all parameters in $\boldsymbol{\psi}_{\text{fg}}$ and $\boldsymbol{\psi}_{\text{oc}}$ could be estimated simultaneously in a single run of the MH algorithm, we choose to separate the parameter estimation into two stages for speed. Foreground parameters $\boldsymbol{\psi}_{\text{fg}}$ are estimated first, assuming there is no background occlusion. Next, background parameters $\boldsymbol{\psi}_{\text{oc}}$ are estimated, keeping the foreground parameters $\boldsymbol{\psi}_{\text{fg}}$ fixed. In this way, each stage only requires us to evaluate the forward model for a single facet per moving object, per iteration of the MH algorithm. When estimating foreground parameters, we only compute the response $\mathbf{s}_{\text{fg}}^{n,k}(\boldsymbol{\psi}_{\text{fg}})$ for each proposal; when estimating background parameters, $\mathbf{s}_{\text{fg}}^{n,k}(\widehat{\boldsymbol{\psi}}_{\text{fg}})$ is fixed using already estimated foreground parameters $\widehat{\boldsymbol{\psi}}_{\text{fg}}$ and we only evaluate $\mathbf{s}_{\text{oc}}^{n,k}(\widehat{\boldsymbol{\psi}}_{\text{fg}}, \boldsymbol{\psi}_{\text{oc}})$ at each algorithm iteration. Separating parameter estimation into two problems is justified by the fact that foreground objects and occluded background regions generally affect very different swaths of time bins. Additionally, because foreground objects are generally closer, their measured responses are much larger. Thus, foreground parameters $\boldsymbol{\psi}_{\text{fg}}$ may be accurately estimated without incorporating background occlusion into the model. Although inter-frame parameter priors could be incorporated into our algorithmic framework, we demonstrate good performance with simple uniform priors on the parameters in $\boldsymbol{\psi}_{\text{fg}}$ and $\boldsymbol{\psi}_{\text{oc}}$, choosing wide bounds to exclude extreme and unrealistic scenarios.

In the foreground parameter estimation stage, we use the MH algorithm to draw samples from the posterior distribution:

$$f_{\text{fg}}(\boldsymbol{\psi}_{\text{fg}} \,|\, \mathbf{x}) \propto f_{\text{fg}}(\mathbf{x} \,|\, \boldsymbol{\psi}_{\text{fg}})\, g_{\text{fg}}(\boldsymbol{\psi}_{\text{fg}}), \tag{29}$$

[2]Specifically, we apply the algorithm on pg. 6, which was developed for use on a uniform floor.

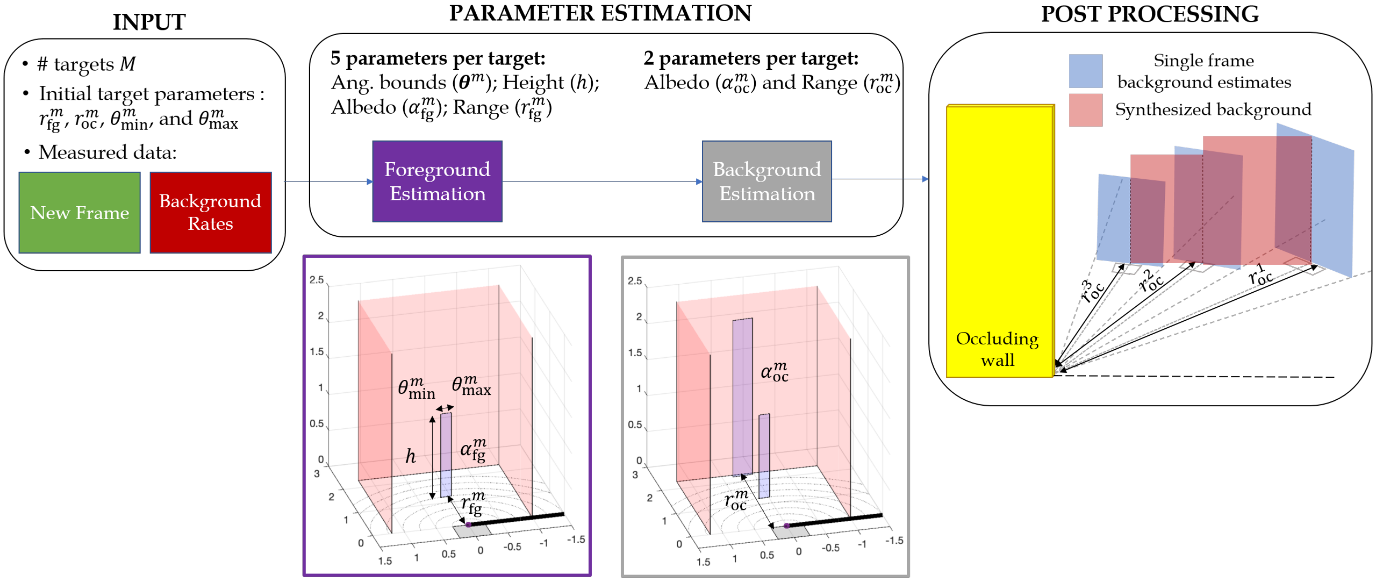

Supplementary Figure 7: Outline of the parameter estimation and post processing algorithm steps.

where $g_{\text{fg}}(\boldsymbol{\psi}_{\text{fg}})$ is the prior on $\boldsymbol{\psi}_{\text{fg}}$,

$$g_{\text{fg}}(\boldsymbol{\psi}_{\text{fg}}) \propto \begin{cases} 1, & \text{if} \quad \boldsymbol{\psi}_{\text{fg}} \in \mathbf{S}_{\text{fg}}; \\ 0, & \text{otherwise}, \end{cases} \tag{30}$$

with $\mathbf{S}_{\text{fg}}$ defined to be the set of all possible values of $\boldsymbol{\psi}_{\text{fg}}$ that arises from uniform priors on each of the parameters in $\boldsymbol{\psi}_{\text{fg}}$. The likelihood $f_{\text{fg}}(\mathbf{x} \,|\, \boldsymbol{\psi}_{\text{fg}})$ of measurement $\mathbf{x}$ given parameter $\boldsymbol{\psi}_{\text{fg}}$ may be approximated as

$$f_{\text{fg}}(\mathbf{x} \,|\, \boldsymbol{\psi}_{\text{fg}}) \approx \prod_{n=1}^{N} \prod_{k=1}^{K} \frac{\left(\mathbf{b}^{n,k} + \mathbf{s}^{n,k}_{\text{fg}}(\boldsymbol{\psi}_{\text{fg}})\right)^{\mathbf{x}^{n,k}} \exp\left(-\left(\mathbf{b}^{n,k} + \mathbf{s}^{n,k}_{\text{fg}}(\boldsymbol{\psi}_{\text{fg}})\right)\right)}{\mathbf{x}^{n,k}!}, \tag{31}$$

where the effects of background occlusion have been excluded from the model for speed. Candidate parameter values $\boldsymbol{\psi}'_{\text{fg}}$ are drawn according to $\boldsymbol{\psi}'_{\text{fg}} \sim \mathcal{N}(\boldsymbol{\psi}^t_{\text{fg}}, \boldsymbol{\Sigma}_{\text{fg}})$, where $\boldsymbol{\psi}^t_{\text{fg}}$ is a vector containing the current state of each unknown parameter and $\boldsymbol{\Sigma}_{\text{fg}}$ is a diagonal matrix with an entry corresponding to each parameter's proposal variance. This matrix is scaled every 100 iterations to achieve an acceptance rate near 23% [7].

Following the MH algorithm, proposal $\boldsymbol{\psi}'_{\text{fg}}$ is accepted with probability ω:

$$\omega = \min\left[1, \frac{f_{\text{fg}}(\mathbf{x} \,|\, \boldsymbol{\psi}'_{\text{fg}}) g_{\text{fg}}(\boldsymbol{\psi}'_{\text{fg}})}{f_{\text{fg}}(\mathbf{x} \,|\, \boldsymbol{\psi}^t_{\text{fg}}) g_{\text{fg}}(\boldsymbol{\psi}^t_{\text{fg}})}\right] \tag{32}$$

$$\overset{(a)}{=} \begin{cases} \min\left[1, \frac{f_{\text{fg}}(\mathbf{x} \,|\, \boldsymbol{\psi}'_{\text{fg}})}{f_{\text{fg}}(\mathbf{x} \,|\, \boldsymbol{\psi}^t_{\text{fg}})}\right], & \text{if} \quad \boldsymbol{\psi}_{\text{fg}} \in \mathbf{S}_{\text{fg}}; \\ 0, & \text{otherwise}, \end{cases} \tag{33}$$

where (a) arises from the fact that for $\boldsymbol{\psi}^t_{\text{fg}}$ to have been accepted, $g_{\text{fg}}(\boldsymbol{\psi}^t_{\text{oc}}) > 0$, so that

$$\frac{g_{\text{fg}}(\boldsymbol{\psi}'_{\text{fg}})}{g_{\text{fg}}(\boldsymbol{\psi}^t_{\text{fg}})} = \begin{cases} 1, & \text{if} \quad \boldsymbol{\psi}_{\text{fg}} \in \mathbf{S}_{\text{fg}} \\ 0, & \text{otherwise.} \end{cases} \tag{34}$$

For each proposal $\boldsymbol{\psi}'_{\text{fg}}$, we evaluate (31) with

$$\begin{aligned}\frac{f_{\text{fg}}(\mathbf{x}\,|\,\boldsymbol{\psi}'_{\text{fg}})}{f_{\text{fg}}(\mathbf{x}\,|\,\boldsymbol{\psi}^t_{\text{fg}})} &= \exp\left[\log\left(\frac{f_{\text{fg}}(\mathbf{x}\,|\,\boldsymbol{\psi}'_{\text{fg}})}{f_{\text{fg}}(\mathbf{x}\,|\,\boldsymbol{\psi}^t_{\text{fg}})}\right)\right] \\ &= \exp\left[\log\big(f_{\text{fg}}(\mathbf{x}\,|\,\boldsymbol{\psi}'_{\text{fg}})\big) - \log\big(f_{\text{fg}}(\mathbf{x}\,|\,\boldsymbol{\psi}^t_{\text{fg}})\big)\right] \\ &= \exp\left[\sum_{n,k}\log\big(f_{\text{fg}}(\mathbf{x}^{n,k}\,|\,\boldsymbol{\psi}'_{\text{fg}})\big) - \sum_{n,k}\log\big(f_{\text{fg}}(\mathbf{x}^{n,k}\,|\,\boldsymbol{\psi}^t_{\text{fg}})\big)\right],\end{aligned} \tag{35}$$

where to prevent a computing overflow, we compute $\log\big(f_{\text{fg}}(\mathbf{x}^{n,k}\,|\,\boldsymbol{\psi}_{\text{fg}})\big)$ as

$$\log\big(f_{\text{fg}}(\mathbf{x}^{n,k}\,|\,\boldsymbol{\psi}_{\text{fg}})\big) = \mathbf{x}^{n,k}\log\Big(\mathbf{s}^{n,k}_{\text{fg}}\Big) - \mathbf{s}^{n,k}_{\text{fg}} - \log\big(\Gamma(\mathbf{x}^{n,k}+1)\big), \tag{36}$$

where $\Gamma(\cdot)$ is the Gamma function. An approximate constrained maximum likelihood (ML) estimate $\widehat{\boldsymbol{\psi}}_{\text{fg}}$ is formed by drawing samples from $f_{\text{fg}}(\boldsymbol{\psi}_{\text{fg}}\,|\,\mathbf{x})$ using the procedure outlined above, binning samples of each parameter in $\boldsymbol{\psi}_{\text{fg}}$ into histograms, and taking the center of the most commonly occurring bin as the estimate of that parameter.

In the background estimation step, we fix the foreground parameter estimate $\widehat{\boldsymbol{\psi}}_{\text{fg}}$ and estimate the parameters $\boldsymbol{\psi}_{\text{oc}}$ that describe the occluded regions behind them. The posterior distribution of $\boldsymbol{\psi}_{\text{oc}}$ given measurement $\mathbf{x}$ is

$$f_{\text{oc}}(\boldsymbol{\psi}_{\text{oc}}\,|\,\mathbf{x}) \propto f_{\text{oc}}(\mathbf{x}\,|\,\boldsymbol{\psi}_{\text{oc}})g_{\text{oc}}(\boldsymbol{\psi}_{\text{oc}}), \tag{37}$$

where $g_{\text{oc}}(\boldsymbol{\psi}_{\text{oc}})$ is the prior on $\boldsymbol{\psi}_{\text{oc}}$:

$$g_{\text{oc}}(\boldsymbol{\psi}_{\text{oc}}) \propto \begin{cases}1, & \text{if } \ \boldsymbol{\psi}_{\text{oc}} \in \mathbf{S}_{\text{bg}};\\ 0, & \text{otherwise,}\end{cases} \tag{38}$$

and $\mathbf{S}_{\text{bg}}$ is the set of possible parameter values that arises from uniform priors on the parameters in $\boldsymbol{\psi}_{\text{oc}}$ and from a constraint that that any parameter $\boldsymbol{\psi}_{\text{oc}}$ must yield positive rates: $\mathbf{b}^{n,k} + \mathbf{s}^{n,k}_{\text{fg}}(\widehat{\boldsymbol{\psi}}_{\text{fg}}) - \mathbf{s}^{n,k}_{\text{oc}}(\widehat{\boldsymbol{\psi}}_{\text{fg}}, \boldsymbol{\psi}_{\text{oc}}) > 0$. With the estimated foreground rates $\widehat{\boldsymbol{\psi}}_{\text{fg}}$ fixed, the likelihood $f_{\text{oc}}(\mathbf{x}\,|\,\boldsymbol{\psi}_{\text{oc}})$, including the effects of occlusion within the hidden scene, is approximately

$$f_{\text{oc}}(\mathbf{x}\,|\,\boldsymbol{\psi}_{\text{oc}}) \approx \prod_{n=1}^{N}\prod_{k=1}^{K}\frac{\Big(\mathbf{b}^{n,k} + \mathbf{s}^{n,k}_{\text{fg}}(\widehat{\boldsymbol{\psi}}_{\text{fg}}) - \mathbf{s}^{n,k}_{\text{oc}}(\widehat{\boldsymbol{\psi}}_{\text{fg}}, \boldsymbol{\psi}_{\text{oc}})\Big)^{\mathbf{x}^{n,k}}\exp\Big(-\Big(\mathbf{b}^{n,k} + \mathbf{s}^{n,k}_{\text{fg}}(\widehat{\boldsymbol{\psi}}_{\text{fg}}) - \mathbf{s}^{n,k}_{\text{oc}}(\widehat{\boldsymbol{\psi}}_{\text{fg}}, \boldsymbol{\psi}_{\text{oc}})\Big)\Big)}{\mathbf{x}^{n,k}!}. \tag{39}$$

Using a procedure similar to the foreground parameter estimation step, we draw samples from the posterior distribution in (37) using the MH algorithm and form an approximate constrained ML estimate $\widehat{\boldsymbol{\psi}}_{\text{bg}}$.

Laser power correction. In our experiments, we observed the laser power to fluctuate over the duration of our acquisitions. Before processing the data, we estimate a multiplicative scaling factor to adjust for any laser power fluctuation that occurred between the reference measurement and the motion frame. Take $\mathbf{b}'$ to be the estimated background rates, before correcting for variable laser power. The motion frame measurement at time t is given by $\mathbf{x}_t$. Assuming that reference and motion frames should have the same rates at close range (because only more distant parts of the scene are changing), we compute the scale factor κ using all N camera pixels summed over the first 10 time bins:

$$\kappa = \frac{\displaystyle\sum_{k=1}^{10}\sum_{n=1}^{N}\mathbf{x}^{n,k}_t}{\displaystyle\sum_{k=1}^{10}\sum_{n=1}^{N}[\mathbf{b}']^{n,k}}. \tag{40}$$

The corrected background measurement, used in all previous parts of this section, becomes $\mathbf{b} = \kappa\mathbf{b}'$.

Combining estimates from subsequent frames

As frames accumulate, the sequence of estimates $\widehat{\boldsymbol{\psi}}_{\rm fg}$ and $\widehat{\boldsymbol{\psi}}_{\rm bg}$ may be processed together to form a reconstruction of the hidden scenery behind the moving objects. This idea is illustrated in the POST PROCESSING box in Supplementary Figure 7. Here, the estimated occluded regions for three subsequent frames are shown in blue. In our post-processing step, we connect the vertical lines running through the center of these facets to form the combined reconstruction shown in red. The height of each red facet is the same as the blue facet from the previous frame. Combined reconstruction results shown in Supplementary Note 4 were produced in this way.

Computing the vertices of occluded regions

An occluded background region is described by range $r_{\rm oc}$ measured from the occluding edge at angle $\theta_{\rm mid} = (\theta_{\rm max} + \theta_{\rm min})/2$. We describe the occluded background region as a subset of the vertical plane facing the occluding edge at range $r_{\rm oc}$, determined by the position and size of the foreground object as well as the location of the laser spot $\mathbf{p}_{\rm l}$.[3] We seek the vertices of the occluded background region so that its response $\mathbf{s}_{\rm oc}$ may be computed using Algorithm 1. Although in certain geometries the occluded background region may not be exactly rectangular, we make a rectangular approximation for speed.

Assume we have chosen our coordinate system so that laser spot $\mathbf{p}_{\rm l}$ is at the origin and the ground is in the xy-plane. The vertical plane that contains the occluded background region may be written in terms of a surface normal vector $\mathbf{n}_{\rm plane} = [\mathbf{n}^x_{\rm plane},\, \mathbf{n}^y_{\rm plane},\, 0]^\top$ and a point on the plane surface $\mathbf{p}_{\rm plane} = [\mathbf{p}^x_{\rm plane},\, \mathbf{p}^y_{\rm plane},\, \mathbf{p}^z_{\rm plane}]^\top$:[4]

$$\mathbf{n}^x_{\rm plane}(x - \mathbf{p}^x_{\rm plane}) + \mathbf{n}^y_{\rm plane}(y - \mathbf{p}^y_{\rm plane}) = 0. \tag{41}$$

A vertex of the background facet $\mathbf{v}_{\rm bg}$ may be written in spherical coordinates:

$$\mathbf{v}_{\rm bg} = \begin{bmatrix} r\cos\alpha\sin\delta \\ r\sin\alpha\sin\delta \\ r\cos\delta \end{bmatrix}, \tag{42}$$

where α is an angle measured down from the positive z-axis, δ is an angle measured from the positive x-axis towards the positive y-axis, and r is the range from the origin. Because the occluded region is projected from the foreground facet onto the back plane, α and δ may be computed using the corresponding vertex $\mathbf{v}_{\rm fg}$ of the foreground facet:

$$\alpha = \tan^{-1}\frac{\mathbf{v}^y_{\rm fg}}{\mathbf{v}^x_{\rm fg}}, \tag{43}$$

$$\delta = \cos^{-1}\frac{\mathbf{v}^z_{\rm fg}}{\|\mathbf{v}_{\rm fg}\|_2}. \tag{44}$$

The unknown range r of the background vertex $\mathbf{v}_{\rm bg}$ is found by substituting the coordinate expressions of (42) into (41) and solving for r:

$$r = \frac{\langle \mathbf{n}_{\rm plane},\, \mathbf{p}_{\rm plane}\rangle}{\mathbf{n}^x_{\rm plane}\cos\alpha\sin\delta + \mathbf{n}^y_{\rm plane}\sin\alpha\sin\delta}. \tag{45}$$

The background vertex corresponding to foreground vertex $\mathbf{v}_{\rm fg}$ is found by substituting (43), (44), and (45) into (42).

[3] If the camera pixel and laser spot are not co-located, the camera pixel has a different occluded background region associated with it. In our model, we account for both. The occluded region due to the pixel view is assumed constant across all pixels and is computed for the center of the camera FOV.

[4] Plane point $\mathbf{p}_{\rm plane}$ and surface normal $\mathbf{n}_{\rm plane}$ may be written in terms of parameters $\boldsymbol{\theta}$ and $\boldsymbol{\psi}_{\rm oc}$.

Supplementary Note 3: Experimental Considerations

In this section, we elaborate on aspects of our experimental setup, including laser and SPAD locations and acquisition times. We will explain how these design choices, made to emulate more modern hardware or simplify our proof of concept experiment, do not change the fundamental concepts that have been demonstrated.

Acquisition Time Analysis

In this work, we use *integration time* to refer to the total time over which the camera collects meaningful data. We use *acquisition time* to refer to the total time it takes the camera to collect, accumulate, and transfer data. Results have been reported in terms of integration time because it reflects the true capability of our proposed imaging system and not certain limitations of our hardware. Newer SPAD arrays are capable of achieving acquisition time roughly equal to the integration time, exploiting multi-gates approaches, such as the one implemented in the 32×32 pixel array presented in [8].

For the older camera [9] used in our experiments, the USB 2.0 standard is used to transfer data from the camera to our lab computer, resulting in the loss of 83% of the measured frames; with the newer USB 3.0 standard, no measured frames would be lost. The limited gating capabilities of our older camera only allow us to observe 800 ns of each frame of duration 10 μs, resulting in the loss of 92% of the illumination periods. Combined, these two acquisition and data-transfer limitations cause our acquisition times to be about $\frac{1}{(0.08)(0.17)} \approx 75$ times longer than they would be on a newer camera.

Although we demonstrate integration times short enough to track objects in motion, the required integration time could be further reduced by increasing the laser power or the SPADs' fill factor and photon detection probability, for instance exploiting 3D stacking technologies or microlens arrays. In a representative measurement in our system, after removing 39 hot pixels, we found that the remaining pixels had detections in approximately 0.0025% of illumination periods (see Supplementary Table 1 in Supplementary Note 4). Thus, we could increase our system detection rates (through some combination of increases of laser power, fill factors, and detection probability) by a factor of ~ 2000 (decreasing our required integration time by the same factor) before reaching the 5% threshold at which dead time distortions are conventionally regarded to become significant [10]. By this calculation, the results in the paper produced using an integration time of 0.4 s could be achieved with 200 μs integration time, enabling tracking at 5000 updates per second. Furthermore, methods to mitigate dead time effects would facilitate interpretation of data at higher count rates [11–13]. Note, however, that we are not considering the computational demands of very high-speed tracking.

Laser location

Although illustrations of our imaging system (Figures 1A and 4A in the main document) depict the laser spot as being in a specific location, our proposed NLOS imaging method is robust to a wide variety of laser positions, assuming the laser spot position is known and updated in the model. This allows the laser itself to be located in a variety of positions on the visible side of the wall.

In Supplementary Figure 8, a bird's eye view of the imaging scenario is used to illustrate some of the trade-offs between different laser spot positions, given that the laser itself has a fixed position. Here, the laser spot is at angle χ measured around the edge into the visible side. In this laser spot position, although a small wedge of the hidden scene (red) is not illuminated by the pulsed laser and is thus not recoverable, the remaining majority of the hidden volume is recoverable using our algorithm. In order to image more of the hidden scene with the same laser position, the laser spot might be directed towards the 'Alternative position' marked in Supplementary Figure 8. While this laser spot illuminates more of the hidden scene, the laser spot is farther from the laser itself and certain parts of the hidden scene resulting in lower signal power. Thus, as long as the laser has an unobstructed view of the floor on the visible side, a laser spot may be chosen to balance the imaging needs of a given application.

With our imaging system robust to a wide variety of laser spot positions, we chose a laser spot position for our experiment that allows us to emulate a SPAD camera with hard gating capability. The SPAD camera available for this project [9] lacked the hard gating capability of many newer SPAD camera models [14, 15]. SPAD pixels experience a period of 'dead time' after detecting a photon, during which no new photons can be detected. Hard gating allows the user to 'turn-on' the pixels at times/ranges of interest. Without hard gating, our measurements are dominated by the strong ballistic contribution (first bounce) of light reflected off the floor, with the subsequent dead time 'hiding' later returning photons from ranges of interest in our hidden scene. In our experimental setup, we emulate hard gating by shining the laser through a hole in the occluding wall and directing the laser spot at the floor on the *hidden* side of the occluding wall, as shown in the first panel of Supplementary Figure 9. The occluding wall then mechanically blocks the first bounce from returning to our SPAD FOV, leaving the SPAD pixels sensitive to light returning later from the hidden scene. As shown in the second panel of Supplementary Figure 9, two mirrors on the hidden side of the occluding wall direct the laser spot closer to the occluding edge and the visible side. Although the laser spot in our experiment is technically on the hidden side of the occluding wall, it does not change the concept being demonstrated. The laser spot position

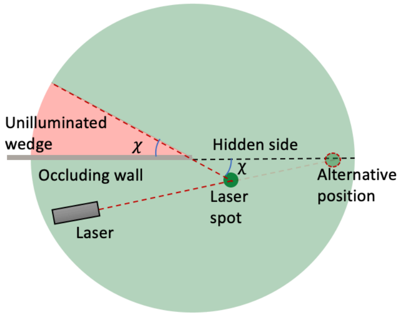

Supplementary Figure 8: A bird's eye view of the occluding wall and a possible laser spot position. Here, the laser spot is at an angle χ measured around the edge into the visible scene. At this angle, the small wedge shown in red is not illuminated by the laser and thus not recoverable by our algorithm. The alternative position illuminates more of the hidden scene but with more signal attenuation for certain parts of the hidden scene due to greater distances between bounces.

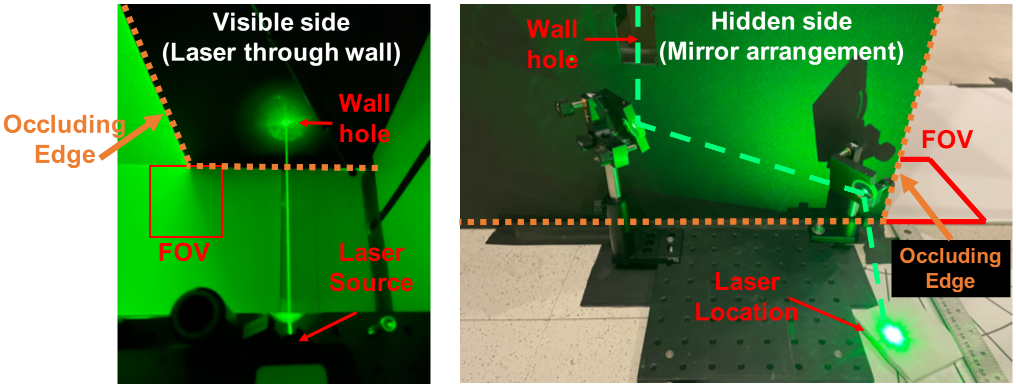

Supplementary Figure 9: Emulating the hard gating ability of the SPAD array punching a hole through the wall. Two mirrors are set-up on the hidden side of the room to redirect the laser close to the vertical edge.

in our experiment illuminates no more of the hidden scene than the alternative position marked in Supplementary Figure 8. A practical realization of our system with newer hard-gated camera hardware would *not* require a hole in the wall.

SPAD location

For the sake of experimental simplicity, our experimental results were achieved using a SPAD array mounted on a tripod and pointed straight down at the FOV on the floor. The setup shown in Figure 1A of the main document, where the camera views the same swath of floor but from a different angle, can be realized when differences in projected pixel area are included in the model.

Supplementary Note 4: Additional Experimental Results

Our inversion algorithm has been tested in a variety of conditions, including different hidden scenes, lighting conditions, and frame lengths. In this note, we first demonstrate our reconstruction algorithm on a hidden scene containing a single, moving planar object and explore the effects of frame length and reference measurement integration time. Then, we show more detailed reconstruction results for the scene containing two moving planar objects that was presented in the main document. Finally, we demonstrate the robustness of our algorithm through results for scenes with non-planar moving objects, more complicated stationary hidden scenery, and with extreme amounts of ambient light.

Single-object demonstrations

In Supplementary Figure 10, we show reconstruction results for a scenario where a single planar facet, at a range of 1.25 m from the occluding edge, is moved in angle through 14 positions in the hidden scene. Figure columns correspond to frame integration times of 0.8 s, 0.4 s, and 0.14 s. The first two rows show different views of the reconstructed hidden scene. The combined background reconstruction, accumulated over the 14 frames, is shown in blue. The reconstructed foreground facets at all 14 positions are plotted on top of each other in red. The SPAD FOV is shaded light gray, the thick dark line on the floor marks the footprint of the occluding wall, and the dotted arcs mark points on the floor that are 0.5 m, 1 m, 1.5 m, and 2 m from the occluding edge. The third figure row shows sample measured histograms at three different pixels in the camera FOV. Note that in the measured histograms, the peak at about 1 m corresponds to the first bounce. Due to the location of the laser spot, the first bounce is occluded from view for some SPAD pixels and is thus most pronounced in the histograms for other pixels (e.g., in Pixel 1024 of Supplementary Figure 10). The much smaller peak near 3.5 m in range is due to the foreground object, and the counts observed between 3.5 m and 8 m are due to the back walls and ceiling. For all three frame lengths, the foreground facets are correctly placed on a 1.25 m arc around the occluding edge. Foreground facet height estimates are also very close to the true height of 1.1 m. This is easily observed in first figure row, where the tops of reconstructed foreground facets clearly align with each other with very little variability. For all three frame lengths, we observe that the background accuracy is greatest at azimuthal angles closer to the hidden side, with increasing error deeper in angle into the hidden scene. As expected, scenery deeper into the hidden scene illuminates fewer camera pixels and thus results in lower measurement SNR. We note that although foreground estimates are quite accurate for all three integration times, error in the background reconstruction increases as the integration time decreases.

In Supplementary Figure 11, we use the same single-object scenario as in Supplementary Figure 10 to explore the effect of reference measurement integration time on reconstruction accuracy. Here, each new frame has an integration time of 0.8 s while testing reference measurement integration times of 8.1 s, 3.3 s, and 1.6 s seconds. Figure columns show results for different integration times; figure rows show two different reconstruction views. As in Supplementary Figure 10, foreground facets are correctly placed in range at 1.25 m. However, when stationary scene integration time is decreased to 1.6 s, we observe more error in foreground facet height estimates. Similarly, background reconstructions for 8.1 s and 3.3 s are comparable to the results in Supplementary Figure 10, where an integration time of 33 s was used for the reference measurement. Increased error is seen in the background reconstruction when background integration time is reduced to 1.6 s. Although some of the results in this paper use the longest available background integration times, the results in Supplementary Figure 11 suggest that there is little cost to using background integration times as short as 3.3 s.

The results in Supplementary Figures 10 and 11 were for a single object fixed in range, and moving in angle around the occluding edge. In Supplementary Figure 12, we fix a object in angle at $\pi/2$ and move it in range away from the occluding edge to demonstrate our algorithm on a variety of object positions within the hidden scene. Each figure column corresponds to a different object range (labeled above). The first row shows LOS photographs of the hidden scene in each case, and the second row shows our corresponding reconstructions. Because the object is not moving in angle, we do not attempt to process multiple frames into a combined background reconstruction. Instead, we plot the single-frame reconstruction results for each object position. Reconstruction results for the first five object positions closely match the ground truth. When the object reaches 1.75 m in range from from the edge, it is extremely close to the back wall and the reconstruction quality starts to degrade slightly, although the ranges of both foreground and background reconstructions are still very accurate. Because parts of the planar facet at 1.75 m share round-trip travel times with the back wall, separately estimating foreground and background parameters is less justified and there is less total change in the measurement due to the object.

Two-object demonstration

In Supplementary Figures 13 and 14, we show more detailed reconstruction results for the two-object scenario in the main document. Columns of Supplementary Figure 13 correspond to seven measurement frames as the objects move towards and then past each other. The object that starts on the left in Frame 1 is fixed at a range of 1 m; the object that starts on the right side is

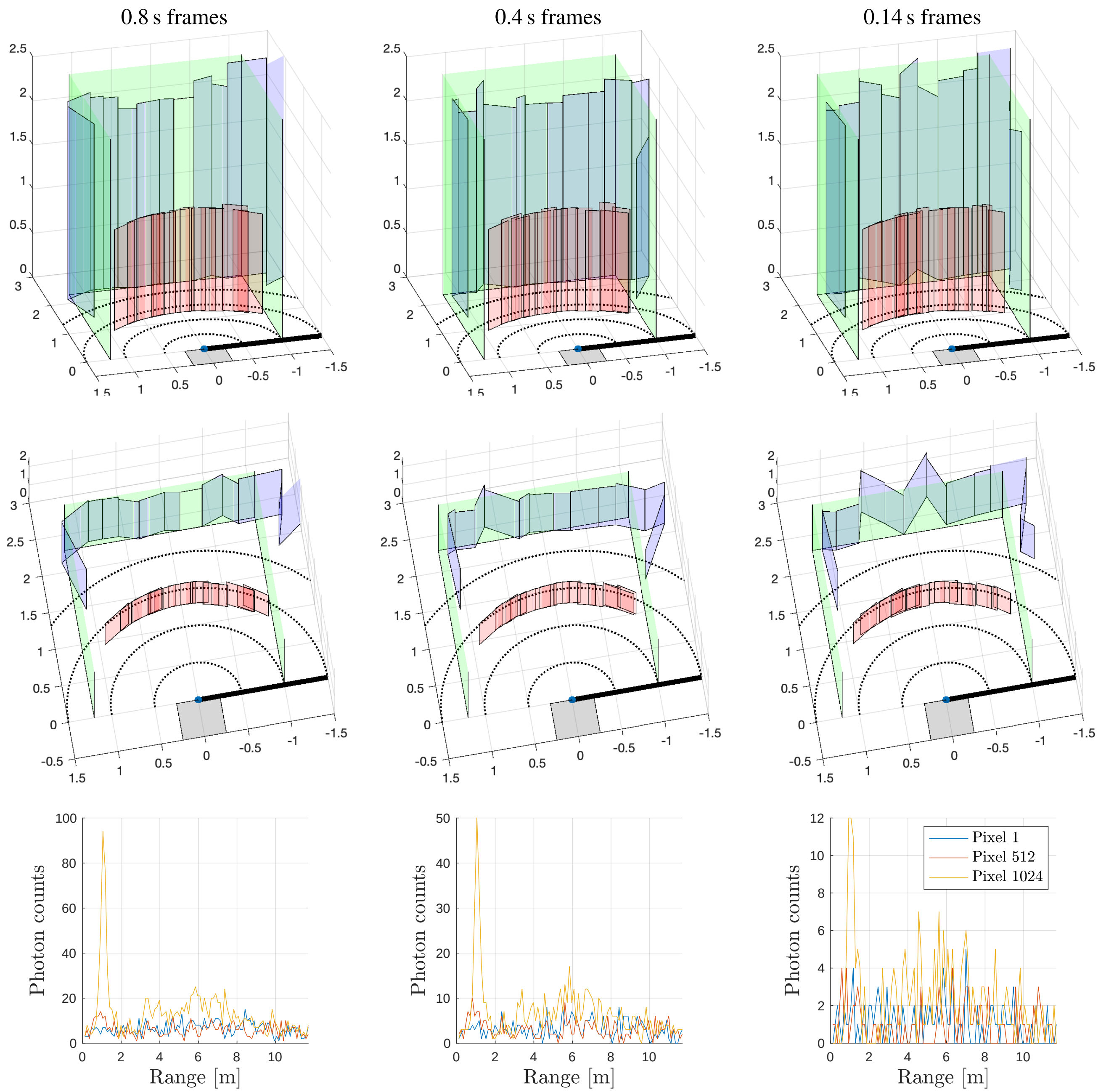

Supplementary Figure 10: Experimental results for when a single planar facet at a range of 1.25 m from the occluding edge moves in azimuthal angle through 14 positions in the hidden scene. Figure columns correspond to three different frame integration times: 0.8 s, 0.4 s, and 0.14 s. The first and second rows show two different views of the reconstructed hidden scene. The true location of the stationary back walls are shown in green for reference. The composite background reconstruction, accumulated over the 14 frames, is shown in blue. The reconstructed foreground facets at all 14 positions are plotted in red. The SPAD FOV is shaded light gray, the thick dark line on the floor marks the footprint of the occluding wall, and the dotted arcs mark points on the floor that are 0.5 m, 1 m, 1.5 m, and 2 m from the occluding edge. The third figure row shows measured histograms at three different SPAD pixels. Results were generated using a reference measurement integrated over 33 s.

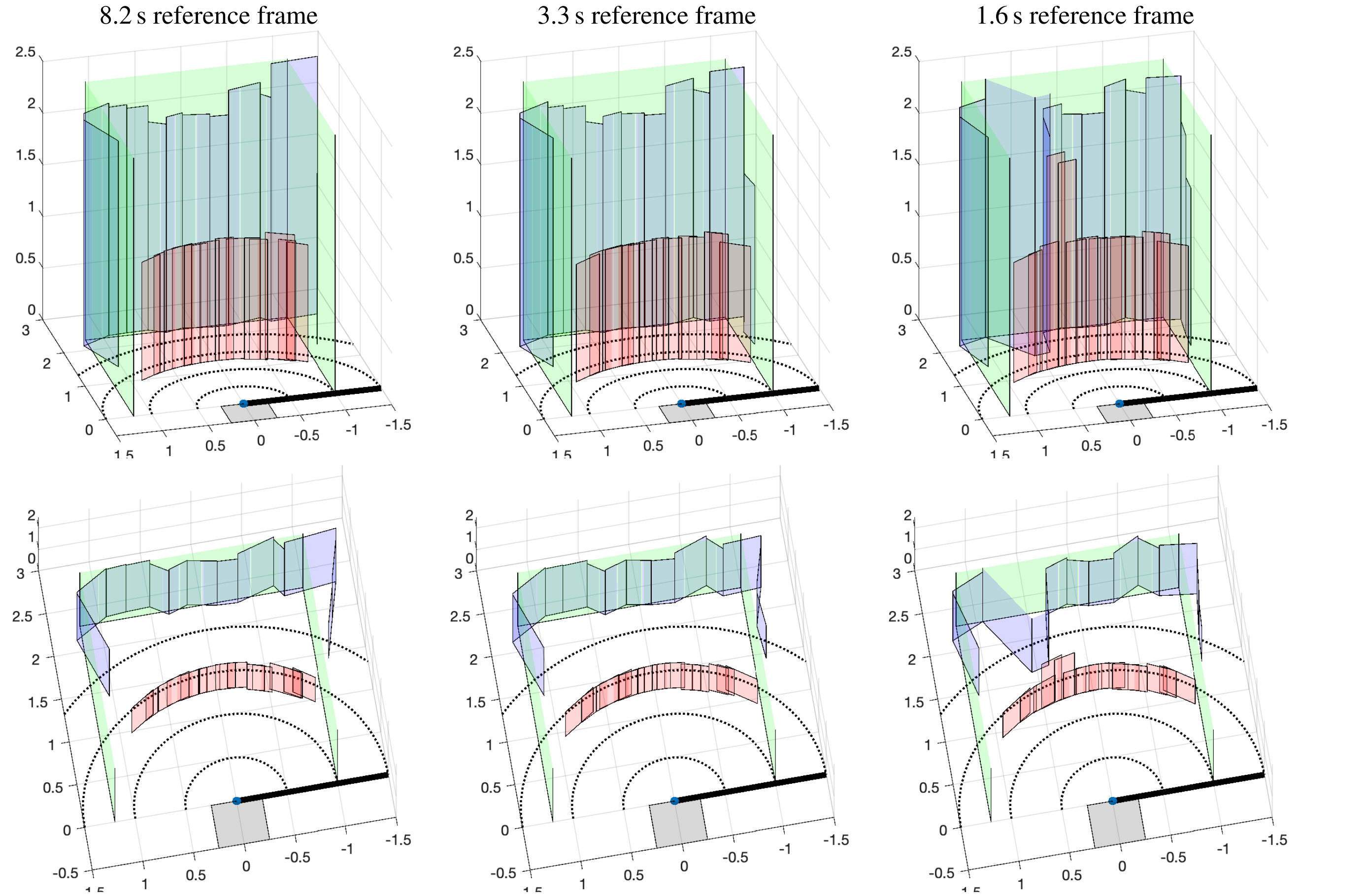

Supplementary Figure 11: Hidden scene reconstructions for reference measurement integration times of 8.2 s, 3.3 s, and 1.6 s. The scenario is the same as in Supplementary Figure 10, with a single planar facet fixed at a range of 1.25 m and swept in angle through 14 positions. Each new measurement frame is integrated over 0.8 s. Figure rows show two different views of the hidden scene reconstruction. The true location of the stationary back walls are shown in green for reference. The composite background reconstruction, accumulated over the 14 frames, is shown in blue. The reconstructed foreground facets at all 14 positions are plotted in red. The SPAD FOV is shaded light gray, the thick dark line on the floor marks the footprint of the occluding wall, and the dotted arcs mark points on the floor that are 0.5 m, 1 m, 1.5 m, and 2 m from the occluding edge.

slightly farther away at 1.25 m. LOS ground truth photographs are shown in the first row, with single-frame reconstruction results shown in the second row. For increased legibility, the z-axis in the reconstructed frames is cropped at 2.5 m, although the true ceiling height is 3 m. In all but Frame 6, two objects are correctly resolved and placed with very little error in range. There is some variability in object height estimates, most notably in Frames 4 and 5. In Frame 6, when the left object begins to cross in front of the right object, our algorithm resolves a single object. In all result frames, the background estimates closely match the ground truth. In Supplementary Figure 14, we show two views of the combined background reconstruction, formed by accumulating a total of 13 background estimates (two each from Frames 1–5 and 7, and one from Frame 6). The estimate (blue) is extremely close to the measured ground truth (green).

Robustness demonstrations

In Supplementary Figure 15, we explore the effects of model mismatch on our inversion algorithm. Each figure column corresponds to a different hidden object type while rows show ground truth LOS photographs and single-frame reconstruction results. The white and gray facet objects fit the rectangular, planar facet model, although the gray facet reflects fewer photons resulting in lower SNR. The mannequin is not a facet at all, and it is meant to test the common scenario where the hidden object is a person moving through the hidden scene. The stairs object is planar, but it is also wide and not rectangular, similar perhaps to a piece of furniture that has been relocated within the hidden scene or a moving car. The white facet reconstruction is the most accurate of the four examples. We note that the gray facet reconstruction is slightly wider than the ground truth (and the white

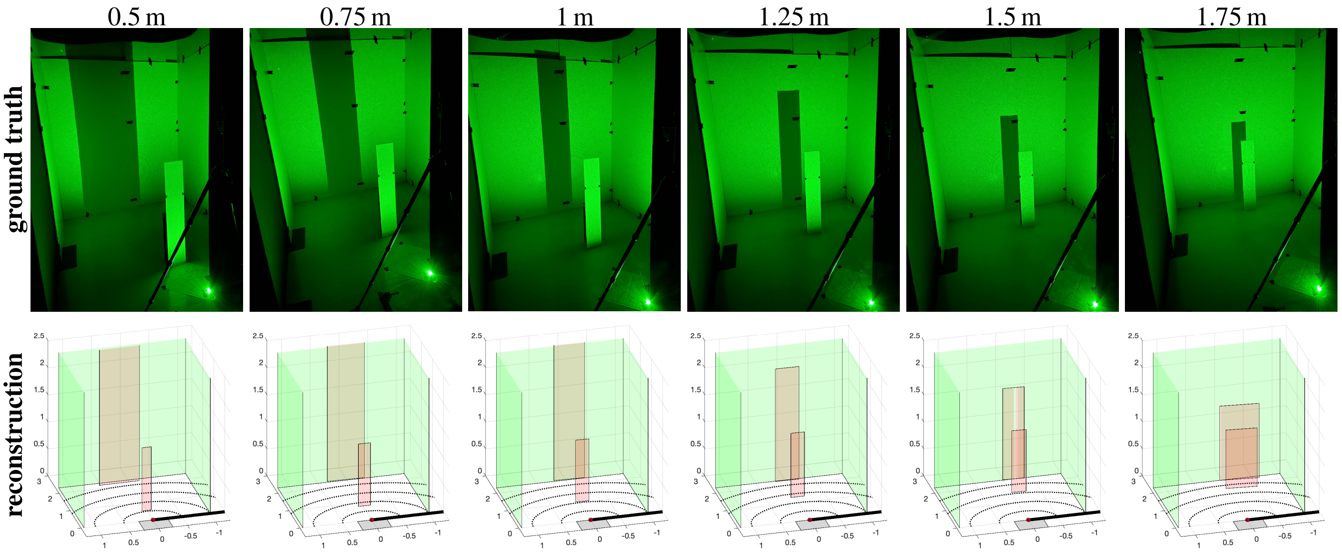

Supplementary Figure 12: Hidden scene reconstructions for a object fixed in angle at $\pi/2$ (measured around the occluding edge and into the hidden scene) and moved through six different positions in range. The first figure row shows a reference LOS photograph of the hidden scene; the second row shows the single-frame reconstruction results. In the reconstructions, the true location of the back wall is shown in green with foreground and background reconstructions shown in red. Foreground and background reconstructions closely match the ground truth in all cases, with more error in the last column when the moving object is very close to the back wall. Each measurement frame had a 0.4 s integration period, with the reference measurement integrated over 32.6 s.

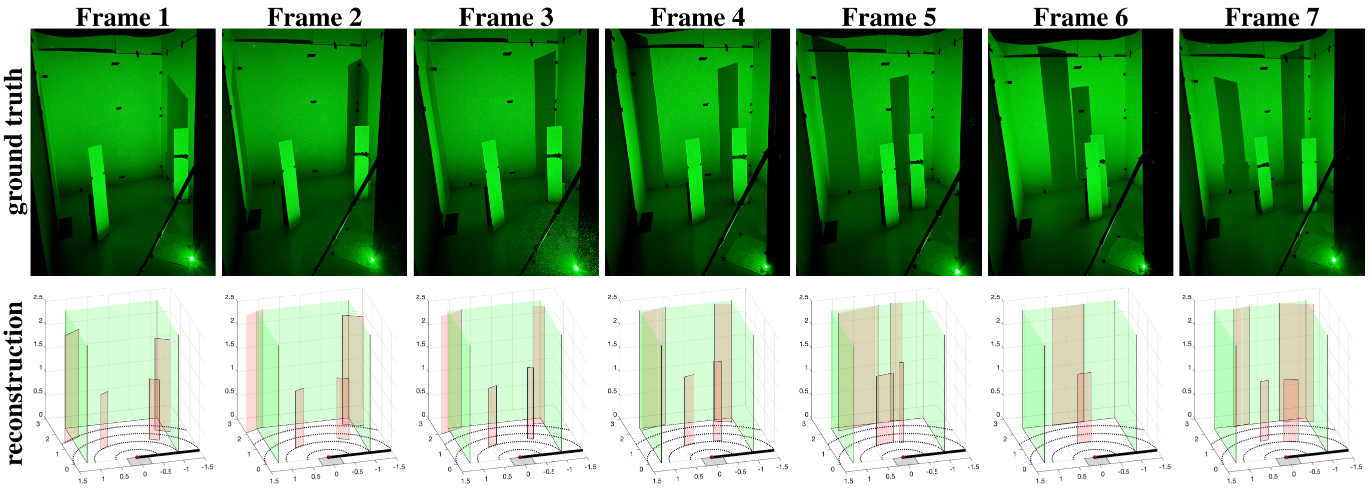

Supplementary Figure 13: Single-frame reconstruction results for seven different frames of a two-object scenario. The first figure row shows LOS photographs of the ground truth; the second row shows single-frame reconstruction results. The true location of the back wall is shown in green with foreground and background reconstructions shown in red. Each measurement frame had a 0.4 s integration period, with the reference measurement integrated over 32.6 s.

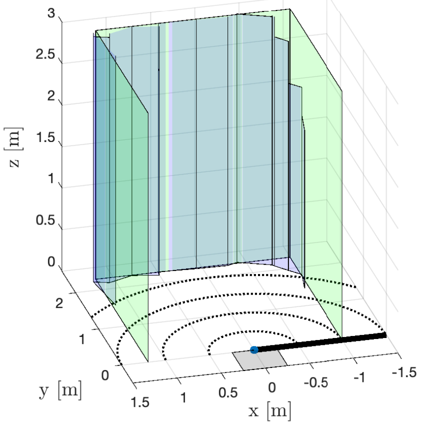

(a) Side view

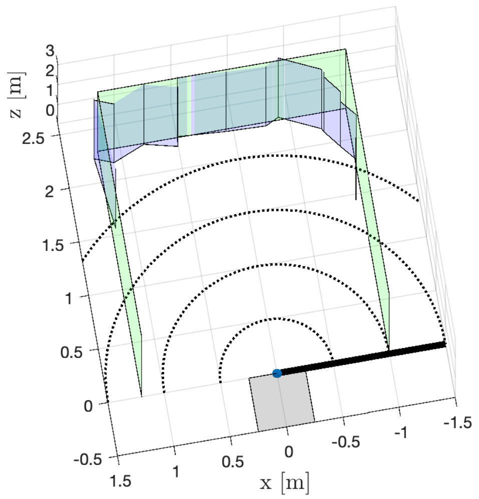

(b) Bird's eye view

Supplementary Figure 14: Two views of the *combined* wall reconstruction formed using the seven two-object frames in Supplementary Figure 13. The true location of the back wall is shown in green with stationary scene reconstructions shown in blue. Each measurement frame had a 0.4 s integration period, with the reference measurement integrated over 32.6 s. (a) side view; (b) bird's eye view.

facet reconstruction), with error likely due to the SNR reduction. In all four cases, the hidden object was fit by a rectangular planar facet placed correctly in range, and the range of the occluded background region is accurately recovered. We note that although our model does not allow us to reconstruct the varying height profile of the stairs, we correctly reconstruct it to be wider and more to the right than the other hidden objects. These results indicate that our rectangular, planar-facet model does not prevent our inversion algorithm from giving useful results under significant model mismatch. Instead, we expect to correctly locate and roughly describe (in width and height) a variety of hidden objects while also reconstructing the hidden scene behind them.

Our reconstruction approach does not require any knowledge of the stationary hidden scene, as long as its *response* can be well characterized by a reference measurement. As a result, our inversion algorithm is not constrained to work only on simple hidden scenes containing just a few walls. In Supplementary Figure 16, we demonstrate our inversion algorithm on a stationary hidden scene that contains a large stationary object in the foreground. In the first figure row, we show LOS photographs of the hidden scene. The first photograph, taken of the stationary scene before the moving object enters, shows a large white foreground object in addition to side and back walls. After the object enters the scene, it remains at an azimuthal angle of $\pi/2$ around the corner and moves to 1 m, 1.25 m, and 1.5 m ranges. At these positions, the moving object shares some round-trip travel times with the large stationary facet. The second figure row shows single-frame reconstruction results for the three moving object positions. In all cases, an accurate reconstruction of both the moving object and the occluded background region is formed.

In Supplementary Figure 17, we evaluate our algorithm with high background counts created by turning the light on in the lab. Supplementary Figure 17a shows a photograph of the entire lab with the overhead lights on, Supplementary Figure 17b shows just the hidden scene, and Supplementary Figure 17c shows the hidden scene once the moving object has entered. Spatially averaged measurements for the reference and motion frames are shown in Supplementary Figure 17d, with their difference shown in Supplementary Figure 17e. Although the reference and motion frames and essentially indistinguishable with the naked eye, their difference, while noisy, has a discernible peak at the range of the moving target and a dip at the range of the occluded background wall; compare with Fig. 4C of the main paper, in which the difference is much less noisy. Single-frame reconstruction results are shown in Supplementary Figure 17f. Despite the unmodeled effects of detector dead time and the large noise variance due to high background counts, the reconstructed object and background closely match the ground truth.

Supplementary Table 1 provides data from representative two-minute experiments with and without overhead lights. The number of integrated frames is not determined entirely by the acquisition time because of the random loss of frames over the USB 2.0 interface. Of the 1024 SPAD pixels, 39 are designated hot. After removal of these pixels, the remaining 985 pixels have about 100 times more photon detections with overhead lights on. The table outlines the computations of counts per pixel per illumination pulse under each of the two conditions. Since dead time effects are negligible, we may approximate the signal-to-ambient ratio for the results in Supplementary Figure 17 as $\frac{0.0000252}{0.00309-0.0000252} \approx 0.008$.

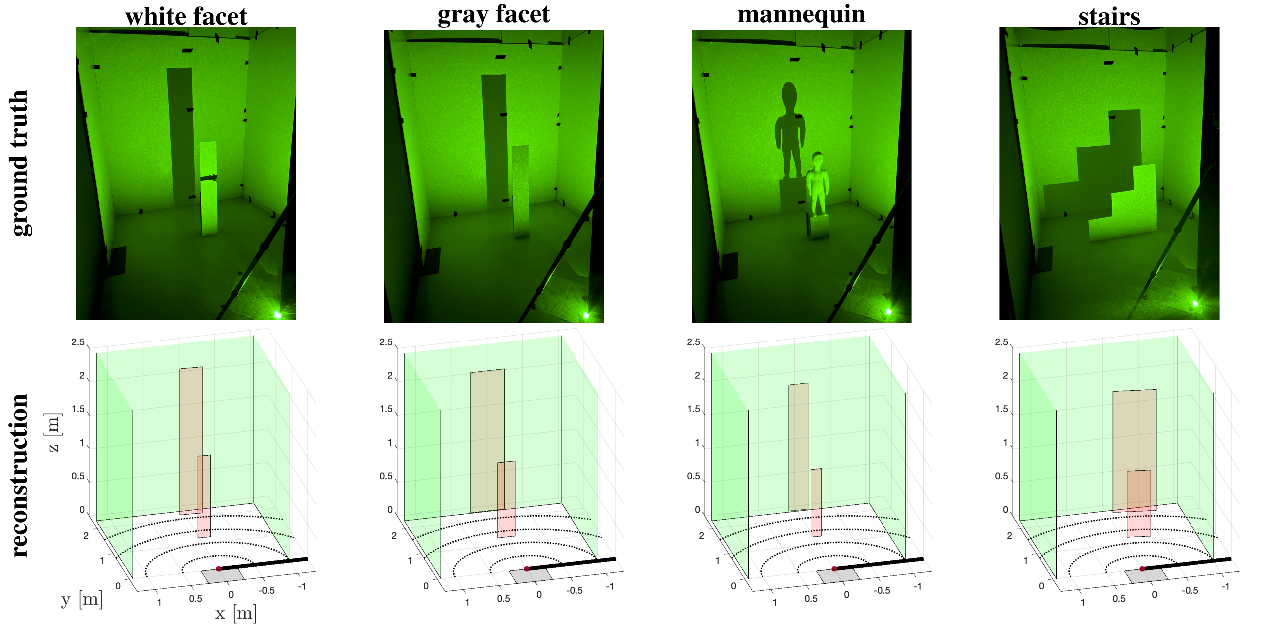

Supplementary Figure 15: A demonstration of algorithm robustness to different object types at a distance of 1.25 m from the occluding edge. The four columns correspond to different object types: a white facet, a dark gray facet, a mannequin, and a stair-shaped facet. The first row shows LOS photographs of the hidden scene; the second row shows our reconstructions. The true location of the back wall is shown in green with foreground and background reconstructions shown in red. Although the first two objects perfectly match the rectangular, planar-facet model, the dark gray facet returns far fewer photons, and the mannequin and stairs are not rectangular facets at all. In all four cases, the foreground object is well fit by our facet model and the occluded background region is placed correctly in range. Each measurement frame had a 0.4 s integration period, with the reference measurement integrated over 33 s.

	**Negligible ambient light**	**Overhead lights**
Integrated frames	2.118×10^6 frames	2.102×10^6 frames
Detections	2.10×10^6 counts	2.56×10^8 counts
Pixels	985	985
Pulses per frame	$\left(50 \times 10^6 \frac{\text{pulses}}{\text{sec}}\right) \left(800 \times 10^{-9} \frac{\text{sec}}{\text{frame}}\right) = 40 \frac{\text{pulses}}{\text{frame}}$	$\left(50 \times 10^6 \frac{\text{pulses}}{\text{sec}}\right) \left(800 \times 10^{-9} \frac{\text{sec}}{\text{frame}}\right) = 40 \frac{\text{pulses}}{\text{frame}}$
Detection rate	$\frac{2.10 \times 10^6 \text{ counts}}{2.118 \times 10^6 \text{ frames}} \left(\frac{1 \text{ frame}}{40 \text{ pulses}}\right) \left(\frac{1}{985 \text{ pixels}}\right) = 0.0000252 \frac{\text{counts}}{\text{pixel} \cdot \text{pulse}}$	$\frac{2.56 \times 10^8 \text{ counts}}{2.102 \times 10^6 \text{ frames}} \left(\frac{1 \text{ frame}}{40 \text{ pulses}}\right) \left(\frac{1}{985 \text{ pixels}}\right) = 0.00309 \frac{\text{counts}}{\text{pixel} \cdot \text{pulse}}$

Supplementary Table 1: Measurements from representative experiments with and without overhead lights, along with derived count rates per laser pulse. Counts from 39 hot pixels are removed.

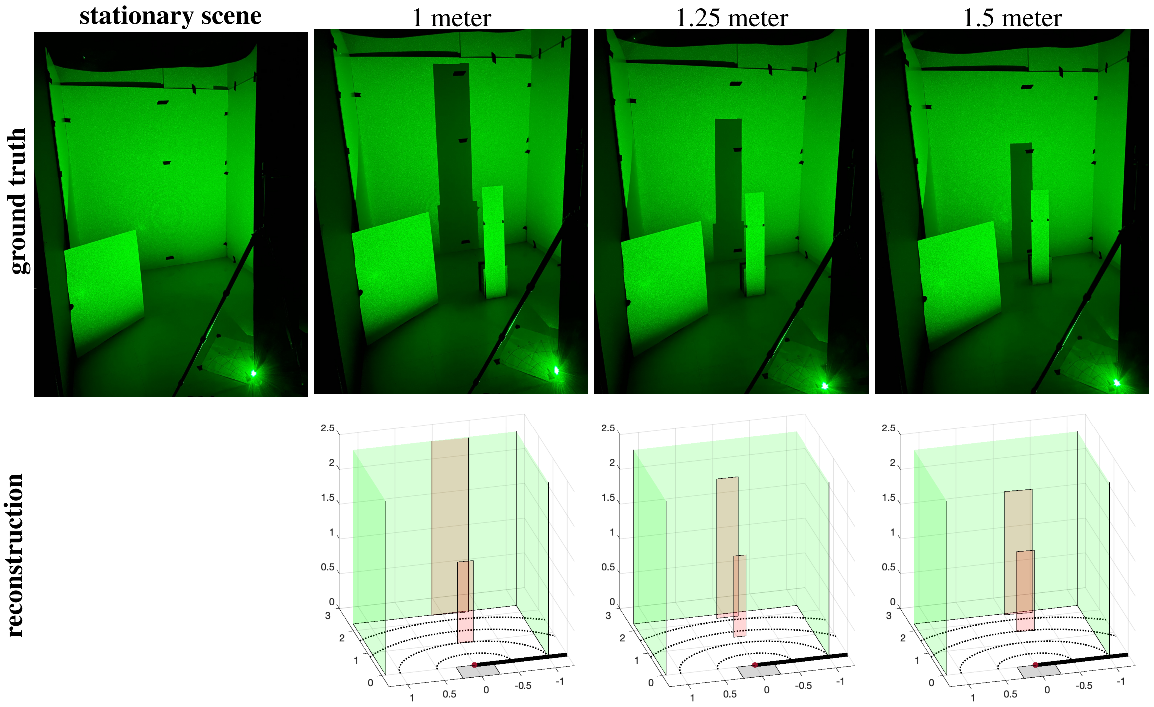

Supplementary Figure 16: Reconstruction results for a challenging hidden scene with a large stationary foreground object. The first row shows LOS photographs of the hidden scene; the second row shows the corresponding reconstructions. The true location of the back wall is shown in green with foreground and background reconstructions shown in red. The first column shows a photograph of the stationary scene, acquired before the moving object enters the hidden scene. Subsequent columns correspond to three different positions of the moving object, which is fixed in azimuthal angle at $\pi/2$ (measured around the edge and into the hidden scene) and moved in range to 1 m, 1.25 m, and 1.5 m from the occluding edge. Note that even though the stationary foreground object and the moving object occupy similar range bins, reconstructions closely match the ground truth in all test cases. These results were produced using a 0.4 s integration time per frame with an 24.5 s reference measurement.

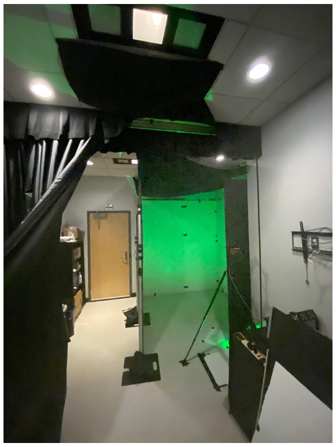

(a) Laboratory photo

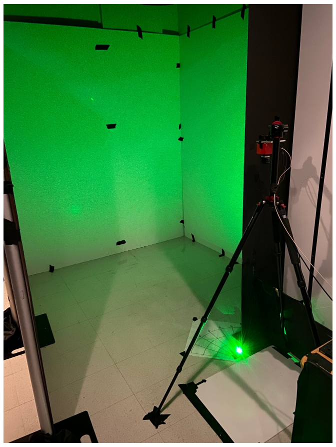

(b) Stationary hidden scene

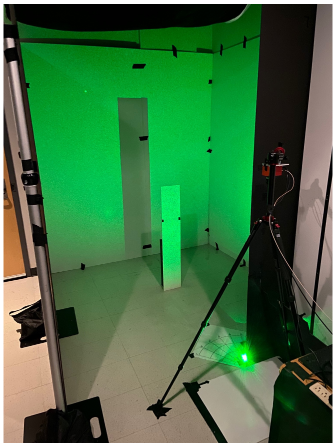

(c) Hidden scene with object

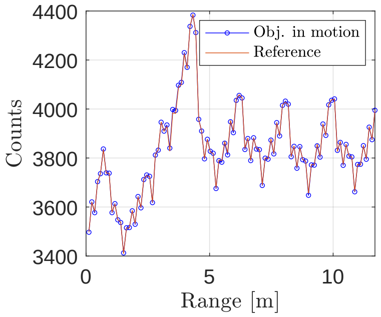

(d) Spatially averaged measurements

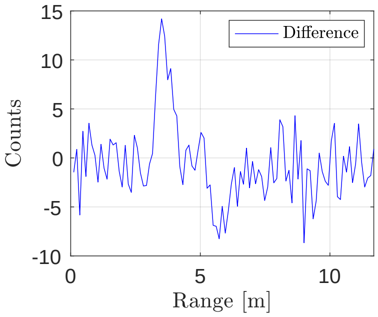

(e) Spatially averaged difference measurement

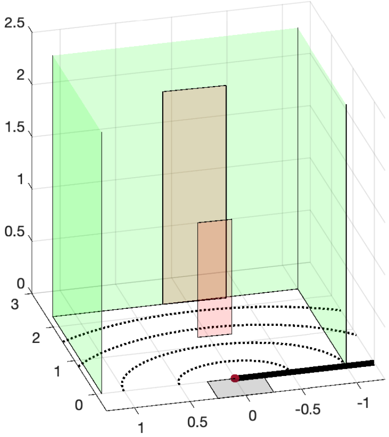

(f) Reconstruction

Supplementary Figure 17: A demonstration of algorithm robustness to the extreme amount of ambient light introduced by turning on the overhead lighting, as shown in (a). An LOS photo of the scene during a reference measurement is shown in (b), and a photo of the hidden scene after an object (at a range of 1.25 m and azimuthal angle of $\pi/2$) has entered the hidden scene is shown in (c). Spatially averaged measurements for the stationary scene (red) and one motion frame (blue) are shown in (d), with their difference shown in (e). Despite the high background counts, the reconstruction in (f) closely matches the ground truth. The results were produced using an integration time of 3.3 s with a reference frame integrated over 24.5 s.

Supplementary References

[1] J. Rapp, C. Saunders, J. Tachella, J. Murray-Bruce, Y. Altmann, J.-Y. Tourneret, S. McLaughlin, R. M. A. Dawson, F. N. C. Wong, and V. K. Goyal, "Seeing around corners with edge-resolved transient imaging," *Nature Communications*, vol. 11, no. 5929, Nov. 2020.

[2] P. P. Klein, "On the ellipsoid and plane intersection equation," *Applied Mathematics*, vol. 3, no. 11, pp. 1634–1640, Nov. 2012.

[3] G. Gariepy, F. Tonolini, R. Henderson, J. Leach, and D. Faccio, "Detection and tracking of moving objects hidden from view," *Nature Photonics*, vol. 10, no. 1, pp. 23–26, 2016.

[4] K. L. Bouman, V. Ye, A. B. Yedidia, F. Durand, G. W. Wornell, A. Torralba, and W. T. Freeman, "Turning corners into cameras: Principles and methods," in *Proc. 23rd IEEE Int. Conf. Computer Vision*, 2017, pp. 2270–2278.

[5] S. W. Seidel, Y. Ma, J. Murray-Bruce, C. Saunders, W. T. Freeman, C. C. Yu, and V. K. Goyal, "Corner occluder computational periscopy: Estimating a hidden scene from a single photograph," in *Proc. IEEE Int. Conf. Computational Photography*, May 2019.

[6] S. W. Seidel, J. Murray-Bruce, Y. Ma, C. Yu, W. T. Freeman, and V. K. Goyal, "Two-dimensional non-line-of-sight scene estimation from a single edge occluder," *IEEE Trans. Computational Imaging*, vol. 7, pp. 58–72, 2021.

[7] A. Gelman, W. R. Gilks, and G. O. Roberts, "Weak convergence and optimal scaling of random walk Metropolis algorithms," *The Annals of Applied Probability*, vol. 7, no. 1, pp. 110–120, 1997.

[8] D. Portaluppi, E. Conca, and F. Villa, "32 × 32 CMOS SPAD imager for gated imaging, photon timing, and photon coincidence," *IEEE J. Selected Topics Quantum Electronics*, vol. 24, no. 2, Mar.–Apr. 2018.

[9] F. Villa, R. Lussana, D. Bronzi, S. Tisa, A. Tosi, F. Zappa, A. Dalla Mora, D. Contini, D. Durini, S. Weyers, and W. Brockherde, "CMOS imager with 1024 SPADs and TDCs for single-photon timing and 3-d time-of-flight," *IEEE J. Selected Topics Quantum Electronics*, vol. 20, no. 6, pp. 364–373, 2014.

[10] D. V. O'Connor and D. Phillips, *Time-correlated Single Photon Counting*. London: Academic Press, 1984.

[11] J. Rapp, Y. Ma, R. M. A. Dawson, and V. K. Goyal, "Dead time compensation for high-flux ranging," *IEEE Trans. Signal Processing*, vol. 67, no. 13, pp. 3471–3486, 1 Jul. 2019.

[12] ——, "High-flux single-photon lidar," *Optica*, vol. 8, no. 1, pp. 30–39, Jan. 2021.

[13] S. Farina, G. Acconcia, I. Labanca, M. Ghioni, and I. Rech, "Toward ultra-fast time-correlated single-photon counting: A compact module to surpass the pile-up limit," *Review of Scientific Instruments*, vol. 92, no. 6, 2021.

[14] M. Renna, J. H. Nam, M. Buttafava, F. Villa, A. Velten, and A. Tosi, "Fast-gated 16 × 1 SPAD array for non-line-of-sight imaging applications," *Instruments*, vol. 4, no. 2, 2020. [Online]. Available: https://www.mdpi.com/2410-390X/4/2/14

[15] S. Riccardo, E. Conca, V. Sesta, and A. Tosi, "Fast-gated 16×16 SPAD array with on-chip 6 ps TDCs for non-line-of-sight imaging," in *Proc. IEEE Photonics Conf.*, 2021, pp. 1–2.
